# Selectived and Reshaped Early Dominant Microbial Community in the Cecum With Similar Proportions and Better Homogenization and Species Diversity Due to Organic Acids as AGP Alternatives Mediate Their Effects on Broilers Growth

**DOI:** 10.3389/fmicb.2019.02948

**Published:** 2020-01-14

**Authors:** Yan Hu, Laidi Wang, Dan Shao, Qiang Wang, Yuanyuan Wu, Yanming Han, Shourong Shi

**Affiliations:** ^1^Poultry Institute, Chinese Academy of Agriculture Sciences, Yangzhou, China; ^2^Center of Effective Evaluation of Feed and Feed Additive, Poultry Institute, Ministry of Agriculture, Yangzhou, China; ^3^Jiangsu Co-innovation Center for Prevention and Control of Important Animal Infectious Diseases and Zoonoses, Yangzhou, China; ^4^Trouw Nutrition R&D, Amersfoort, Netherlands

**Keywords:** organic acids, antibiotic growth promoter alternatives, 16S rDNA sequencing, growth performance, early selective forces of food on microbiome, broilers

## Abstract

Understanding the differences in microbial communities shaped by different food selective forces, especially during early post-hatch period, is critical to gain insight into how to select, evaluate, and improve antibiotic growth promoters (AGPs) alternatives in food animals. As a model system, commercial diet-administered OAs (DOAs) and water-administered OAs (WOAs) were used separately or in combination as Virginiamycin alternatives for broiler feeding during two growth phases: 1–21 days and 22–42 days. Among these three OA-treated groups, the DOA group was most similar to the AGP group in the composition and the proportion of these dominant bacterial communities at the level of phylum, family, and genus in cecal chyme of broilers. Sub-therapeutic Virginiamycin decreased the richness, homogenization, and species diversity of gut microbiota, especially in the early growth stage from days 1 to 21. Among these three OA supplementation schemes, it was clear that DOA supplementation was more likely to increase or maintain the richness, homogenization, species diversity, and predicted gene functions of cecal microbiota in treated broilers than either no supplementation or AGP supplementation during two experimental stages. The interference of DOA treatment with early colonization of probiotics and pathogens in broiler cecum was the most similar to AGP treatment, and OAs did not cause the occurrence of Virginiamycin-resistant strains of *Enterococcus* at the end of this trial. In terms of the predicted gene functions of the microbiota, AGP and DOA treatments provided a similar selective force for microbial metabolism functions in the cecum of broiler chickens, especially in the early growth stage. Noticeably, the relative abundance of some microbiome that was modified by Virginiamycin or DOA supplementation was significantly correlated with body weight gain and KEGG pathway analysis-annotated gene functions such as replication and repair, translation, nucleotide metabolism, and so on. With the comprehensive analysis of these results and practical application, shortened DOA supplementation, after optimization of the amount of addition, would be a suitable alternative to sub-therapeutic Virginiamycin. It was suggested that the programed intestinal microecology under such early selection forces and the effective addition time may be the key elements to focus on the designed alternate strategies of AGPs in food animals.

## Introduction

For a long time, antibiotic growth promoters (AGPs) have been widely used in poultry production to gain weight (Angelakis, 2017) and have shown important agricultural economic benefits as feed additives. However, there has been growing concern about the impact of AGPs, mainly involving the residues of some kinds of antibiotics in poultry produce, the generation and development of antibiotic resistant strains and their migration among intestinal microbiota and to the environment, as well as in humans (Salaheen et al., 2017; Muaz et al., 2018). In recent years, with the addition and promulgation in regulations regarding restricting the use of AGPs and the increasing consumer demand for “antibiotic-free” commercial poultry products, there has been an intensified quest for AGPs alternatives or methods of their use (Kuehn, 2014).

Comparing traditional poultry production with organic poultry production, these emerging issues highlight the need for alternative approaches to improve production performance and feed efficiency without supplementing AGP. These are closely related to microbial load of intestine in food animals (Huyghebaert et al., 2011). In the non-pathological stage, intestinal microbial species include a variety of pathogenic bacteria in the resting state of animals that can maintain homeostasis of symbiosis, and these bacteria can colonize and adjust responses to many factors, including stress, treatment with antibiotics, and diet during critical periods of growth and development (Isaacson and Kim, 2012; Frese et al., 2015). Unlike mammals, the embryonic development of poultry is carried out in the eggs out of the maternal environment, and the establishment of intestinal microflora is relatively lack of guidance from the maternal microflora, so the main selection pressure comes from the diet after birth. As a selective force, antibiotic feeding in food animals has presented the greatest impact on the evolution of their intestinal bacteria (Allen and Stanton, 2014; Andersson and Hughes, 2014), and they promote growth by lowering the incidence of mortality of food animals caused by a pathogen attack in the shortest possible time (Gadde et al., 2017; Muaz et al., 2018). It should not be ignored that the animals fed AGPs still had harmful pathogens in their intestines and did not lead infections. The existing dogmas that certain bacteria (e.g., *Lactobacillus*) can be considered simply as beneficial or not had been challenged by emerging evidence, and highlights the importance of considering even strain level bacterial differences, probably due to their individual components/functional activities and interaction (Broom, 2018). Mammals and human medical research has shown that early bacterial colonization and symbiosis, which not just for some pathogenic bacteria, formatted by AGPs feeding in the early growth, may programmatically affect the incidence of some diseases in adult life (Broom, 2017; Korpela and de Vos, 2018). The hypothesis put forward is that the formation of intestinal microecology under such early selection forces may be the root cause of promoting growth of food animals, rather than AGPs simply changing the abundance of some harmful or beneficial pathogens, which leads to a suggestion that AGPs alternatives could present to achieve similar efficacy on some performance as AGPs.

There are many kinds of AGPs used in food animal production, and no single feed additive can completely replace every sub-therapeutic antibiotic supplementation. The author believes that, based on the requirements of green industry and animal welfare as well as the improvement of feeding and management technology, the designed alternate strategies of AGPs should focus less on improving growth performance and more on improving and effectively maintaining the intestinal health of food animals. A qualified AGPs additive should lead in the better biodiversity and safety of the intestinal microbial symbiosis homeostasis induced by it. Among the most studied non-antibiotic additives are organic acids (OAs), which have been used as additives to promote broilers growth performance (Wyatt and Miller, 1985; Patten and Waldroup, 1988) and have the potential to modify the relative abundance of some bacteria to control losses in the performance of broilers raised in the absence of AGPs (Nava et al., 2009; Samanta et al., 2010; Bagal et al., 2016; Polycarpo et al., 2017). As both short-chain OAs (Nava et al., 2009; Goodarzi et al., 2014) and medium-chain OAs (Rodriguez-Lecompte et al., 2012; Oakley et al., 2014), or both monocarboxylic acids (Thompson and Hinton, 1997; Oakley et al., 2014) and polycarboxylic acids (Rodriguez-Lecompte et al., 2012), or both single (Bagal et al., 2016; Yousaf et al., 2017; Jazi et al., 2018) and blends (Rodjan et al., 2018), OAs added to feed (Nguyen et al., 2018) or water (Rodjan et al., 2018) have shown some promising results for affecting some designated intestinal beneficial bacteria and reducing pathogenic bacteria. Nevertheless, few studies have elucidated the effects of OAs on intestinal microbial communities by the sequencing-based technique in chicken.

Virginiamycin was a streptogramin family of antibiotics, which was created as one of effective alternatives to some kinds of antibiotics (such as Penicillin class) to mainly against Gram-positive aerobic bacteria (Allington and Rivey, 2001), including resistant Staphylococcus and resistant *Enterococcus*, and was widely used at sub-therapeutic concentrations as a feed supplement to enhance poultry productivity. To provide a more comprehensive view for the benefit evaluation of AGPs alternatives, in the present study, a Virginiamycin alternative model was built by using two commercially available acidifying additives. These OAs supplemented though feed or/and drinking water were evaluated separately and in combination to explore their respective specific effects on growth performance, immune-related indices, and intestinal microbiota of broilers. The comparison of the difference in microbial classification, abundance, and diversity in broiler cecal chyme by 16S rDNA sequencing formed under different forces may evaluate the effects of AGPs alternatives on intestinal microbial ecology in food animals during different growth stages.

## Materials and Methods

### Feed Regimens in Chick Model

Diet and drinking water supplement experiments in broilers were designed to determine the effects of two commercially available acidifying additives two OA blends [diet-administered OA (DOA) and water-administered OA (WOA), supplied by Trouw Nutrition, Netherlands] in this trial. The DOA, as a food additive, was a synergistic blend of formic acid, acetic acid, propionic acid, and ammonium formate. The WOA, as a drinking water additive, was blends of OAs with their ammonium salts, mainly formic acid, acetic acid, and ammonium formate. The carrier for these two kinds of OA is silica. The doses of two OA blends were based on the recommendations of supplier. Arbor Acres (AA) broiler cocks at the age of 1 day were obtained from a commercial breeding hennery (Jinghai group, Haian, China). The trial was divided into two phases, 1–21 days and 22–42 days. A total of 400 birds with an initial body weight of 49.20 ± 1.34 g were randomly assigned into five groups (eight replicates/group, 10 cocks/replicate floor pen) consisting of a negative control (NC), a positive control (PC), and three treatment groups. Each floor pen was 1.5 m × 1.5 m × 1.8 m, and the litter consisted of wood shavings. The NC group was provided the basal corn-soybean meal pellet diet with no antibiotic supplementation [basal diet (BD) shown in Table 1] and non-supplemented tap water [basal drinking water (BDW)]; the PC group was provided the BD supplemented with 20 mg/kg AGP (supplied by NUTRECO Bio-Chem Co. Ltd., Zhuzhou, China, containing 50% Virginiamycin with starch and dextrine as carriers for stabilization, whereas those in the other groups received the BD supplemented with the same amount of microencapsulated starch and dextrine) and BDW; the DOA treatment group was provided the BD supplemented with DOA [included in the BD at 4 kg per metric ton (0.4%) in the first phase and 3 kg per metric ton (0.3%) in the second phase] and BDW; the WOA treatment group was provided the BD and BDW supplemented with WOA [added in BDW at approximately 1 kg per metric ton (0.1%) (the exact dosage needs to be confirmed according to a titration trial) in the two phases]; and the last mix OA (MOA) treatment group was provided the BD supplemented with DOA as in the DOA group and simultaneously BDW supplemented with WOA as in the WOA group.

**TABLE 1:** Composition and nutrient levels of the basal diet.

**Items**	**Contents**	
	**Starter stage**	**Grower stage**
	**(1–21 days)**	**(22–42 days)**
**Ingredients (air-dry basis, %)**		
Corn, ground	54.00	56.54
Soybean meal	38.12	35.32
Soybean oil	3.40	3.98
Limestone	1.14	0.93
Dicalcium phosphate	1.86	1.80
DL-methionine	0.25	0.24
Lys (98%)	0.15	0.16
Salt	0.40	0.40
Choline chloride (50%)	0.15	0.10
Mineral premix^1^	0.20	0.20
Vitamin premix^2^	0.03	0.03
Corn starch^3^	0.30	0.30
Total	100.00	100.00
**Nutrient levels**		
Metabolizable energy (MJ kg^–1^)	12.35	12.64
Crude protein (%)	21.00	20.00
Calcium (%)	1.01	0.90
Available P (%)	0.45	0.43
D-Lysine (%)	1.15	1.10
Cysteine (%)	0.29	0.28
Methionine (%)	0.50	0.48

*^1^Indicates that Mineral premix for broiler provided per kilogram of diet: Fe 80.00 mg; Cu 8.00 mg; Mn 100.00 mg; Zn 80.00 mg; I 0.70 mg; Se 0.30 mg. ^2^Indicates that Vitamin premix for broilers provided per kilogram of diet: vitamin A (retinyl palmitate), 8000 IU; vitamin D3 (cholecalciferol), 1000 ICU; vitamin E (dl-α-tocopheryl acetate), 20 IU; vitamin K3 (menadione sodium bisulfate complex), 0.50 mg; vitamin B1, 2.00 mg; vitamin B2, 8.00 mg; vitamin B6, 3.50 mg; vitamin B12 (cobalamin), 10.00 μg; niacin, 35.00 mg; calcium pantothenic, 10.00 mg; folic acid, 0.55 mg; biotin, 0.18 mg. ^3^Indicates that diet-administered OA (DOA) will substitute the respective quantity of corn starch when DOA is used.*

### Sampling Collection and Measurements

Growth performance, including body weight, body weight gain, feed consumption, and survival rate, was recorded at the phases of 1–21 days and 22–42 days. The welfare index—foot pad lesion of all birds was scored on both feet and calculated to an average on day 42. A seven-point scoring system was followed, according to the procedure of Shao et al. (2015). Concurrently, eight cocks (one cock per replicate) from each group were selected at the ages of 21 and 42 days. Blood (approximately 2 mL) from eight cocks from each group was collected via the wing vein and placed into coagulation-promoting vacuum tubes, and the serum of per sample was centrifuged for the subsequent detection of blood parameters (Hu et al., 2019). IgM, IgG, and IgA in serum were determined spectrophotometrically using assay kits from Nanjing Jiancheng Institute of Bioengineering (Nanjing, Jiangsu, China). Commercial hemagglutination inhibition (HI) kits (Groundwork Biotechnology Diagnosticate, San Diego, CA, United States) were used to measure the antibody titer for Newcastle Disease (ND) in serum. The thymus, spleen, and bursa of Fabricius (BF) were removed and weighed. The chyme from the muscular stomach, jejunum, and ceca was collected, and the *p*H was measured with a *p*H electrode (InLab 410 pH-Kombinationselektrode; Mettler Toledo GmbH, Germany). The remaining contents from both ceca were thoroughly mixed and stored at −80°C for 16S rDNA amplicon sequencing analysis. Whole layer litter samples distributed at four angles and the center of each floor pen was taken at the age of 21 and 42 days and then mixed (Shao et al., 2015). The welfare index—litter moisture was measured as described previously (Shao et al., 2015).

Statistical analyses were carried out with SPSS software for Windows (V. 22.0, SPSS Inc., Chicago, IL, United States). Differences in the supplementations were tested using one-way analysis of variance (ANOVA) for independent samples. All data are presented as the mean with pooled SEM values. A *P*-value less than 0.05 was considered significant (Hu et al., 2019).

### DNA Extraction and Sequencing Library Construction

Almost 0.5–1.0 g of homogenized cecal chyme of each chick was used. Total genomic DNA was extracted using the EZNA^TM^ Soil DNA kit (D5625-02, Omega Bio-Tek Inc., Norcross, GA, United States) and stored at −20°C. The V4 region of bacterial 16S rRNA was amplified by PCR using the primer pair 515F/806R (Bergmann et al., 2011; Gao et al., 2017). The amplified products containing main fragments of 400–450 bp were extracted and chosen for further analysis (Caporaso et al., 2011; Gao et al., 2017). PCR products were purified using the GeneJET Gel Extraction Kit (Thermo Scientific, Waltham, MA, United States). After Qubit quantitative and library detection, the individually barcoded 16S rDNA amplicons from each sample were pooled and paired-end sequenced on the IonS5^TM^ XL platform at Novogene Bioinformatics Technology Co., Ltd (Beijing, China), and 250-bp paired-end raw reads were generated.

### Quality Filtering and Sequence Analysis

To obtain the V3–V4 hypervariable region of the bacterial 16S rRNA gene, raw read quality was filtered by Cutadapt (V. 1.9.1). The high-quality sequences were acquired and clustered into operational taxonomic units (OTUs) at 97% identity by the Uparse pipeline (V. 7.0.1001) (Caporaso et al., 2010), and the chimera sequences were excluded from OTUs using the Uchime Algorithm (V. 4.2.40) (Edgar et al., 2011). The taxonomic information of these sequences was annotated by RDP Classifier (Wang et al., 2007; Zhang et al., 2018). The alpha and beta diversity and significance of taxonomic differences between samples were estimated by Qiim (V. 1.9.1) as described previously (Lozupone and Knight, 2005; Gao et al., 2017; Zhang et al., 2018).

## Results

### Growth Performance, Tissue Index, Chyme pH, and Serum Indices

Growth performance in chicks, including the body weight at ages of 21and 42 days (*P* = 0.024 and *P* = 0.006, respectively), daily weight gain (*P* = 0.024, *P* = 0.017, and *P* = 0.006, respectively), average daily intake (*P* = 0.026, *P* = 0.001, and *P* = 0.001, respectively) during the 1–21 days, 22–42 days, and 1–42 days phases and feed consumption (*P* = 0.024) during the 1–21 days phase were significantly affected by the various treatments (Table 2). No significant effect (*P* > 0.05) in the experiment was found regarding the survival rate of the broilers during the 1–21 days and 22–42 days phases or for the water content of the litter, tissue (including thymus, spleen and bursa) index, and gut (including muscular stomach, jejunum, and ceca) chyme pH at the ages of 21and 42 days (Tables 2, 3). In addition, the core of the foot pad lesion (*P* > 0.05) at 42 days of age was not affected by the treatments. Among the serum indices relative to the immune response in broilers in this experiment, IgA (*P* < 0.001) and IgG (*P* = 0.017) at the age of 21 days and IgM (*P* < 0.009) at the age of 42 days were affected by the treatments, whereas IgM at the age of 21 days, IgA and IgG at the age of 42 days and antibody titers of ND at the ages of 21 and 42 days were not (Table 4).

**TABLE 2:** Effects of organic acids supplementation on daily weight gain, average daily intake, feed/gain (g/g), survival rate (%), water content of the litter (g/g), and score of foot pad lesion of broilers during two phases in this experiment.

**Items**	**Groups^1^**					**SEM**	***P*-value**
	**NC**	**PC**	**DOA**	**WOA**	**MOA**		
**1–21 days**							
Body weight at 21th days (g)	976.3^b^	1033.3^a^	1017.5^a^	994.1^b^	1009.1^ab^	6.1	0.024
Daily weight gain (g)	44.2^c^	46.8^a^	46.1^ab^	45.0^bc^	45.7^abc^	0.3	0.024
Average daily intake (g)	63.2^b^	67.8^a^	65.0^b^	64.8^b^	64.2^b^	0.5	0.026
Feed per gain^2^ (%)	1.43^ac^	1.40^b^	1.41^bc^	1.44^a^	1.41^bc^	0.01	0.024
Survival rate (%)	98.75^a^	98.75^a^	98.75^a^	100.00^a^	100.00^a^	0.42	0.736
Water content of the litter^3^ at 21th days (%)	16.3^a^	15.2^a^	15.0^a^	16.0^a^	15.7^a^	0.2	0.388
**22–42 days**							
Body weight at 42 days (kg)	3.19^c^	3.54^a^	3.35^b^	3.40^ab^	3.30^bc^	0.03	0.006
Daily weight gain (g)	105.8^b^	118.9^a^	111.5^a^	114.9^ac^	108.1^bc^	1.4	0.017
Average daily intake (g)	176.3^b^	200.7^a^	189.0^a^	190.0^a^	176.6^b^	2.3	0.001
Feed per gain^2^ (%)	1.67^a^	1.70^a^	1.70^a^	1.65^a^	1.63^a^	0.01	0.209
Survival rate (%)	91.25^a^	98.57^a^	95.00^a^	97.5^a^	97.5^a^	1.09	0.374
**0–42 days**							
Daily weight gain (g)	74.7^c^	83.1^a^	78.7^b^	79.8^ab^	77.5^bc^	0.8	0.006
Average daily intake (g)	119.7^c^	134.0^a^	127.0^b^	127.2^ab^	119.6^c^	1.4	0.001
Feed per gain^2^ (%)	1.60^ab^	1.62^a^	1.62^a^	1.60^ab^	1.57^b^	0.01	0.152
Survival rate (%)	91.3^b^	98.6^a^	95.0^ab^	97.5^ab^	97.5^ab^	1.1	0.207
Water content of the litter^3^ at 42th days (%)	20.6^a^	21.5^a^	22.2^a^	21.0^a^	21.4^a^	0.2	0.244
Score of foot pad lesion at 42th days	0.063^a^	0.046^a^	0.091^a^	0.101^a^	0.056^a^	0.012	0.538

*^1^NC, negative control, basal diet and basal drinking water with no antibiotic supplementation; PC, positive control, antibiotics supplementation; DOA, NC plus diet-administered OA supplementation; WOA, NC plus water-administered OA supplementation; MOA, NC plus diet-administered and water-administered OA supplementation. ^2^Feed per gain (percent of the average daily feed intake relative to the daily weight gain). ^3^Water content of the litter (percent of the wet weight minus the dry weight relative to the wet weight). Values are expressed as means with pooled SEM values. *P*-value is expressed combined significance. In the same line, values with different letters are significantly different for all possible combinations of these different groups (*P* < 0.05 or *P* < 0.01).*

**TABLE 3:** Effects of organic acids supplementation on tissues index^1^ relative to immune response and gut chyme *p*H in broilers at the age of 21 and 42 days in this experiment.

**Items**	**Groups^2^**					**SEM**	***P*-value**
	**NC**	**PC**	**DOA**	**WOA**	**MOA**		
**21th days**							
Thymus index (%)	1.87^a^	2.20^a^	1.77^a^	1.95^a^	2.00^a^	0.09	0.659
Spleen index (%)	0.92^a^	1.22^a^	0.99^a^	0.95^a^	1.07^a^	0.04	0.132
Bursa index (%)	1.50^a^	1.69^a^	1.53^a^	1.65^a^	1.53^a^	0.10	0.973
Muscular stomach *p*H	3.15^a^	2.57^a^	2.73^a^	2.42^a^	2.79^a^	0.08	0.055
Jejunum *p*H	5.83^a^	6.02^a^	6.06^a^	6.12^a^	6.00^a^	0.04	0.242
Ceca *p*H	6.69^a^	6.56^a^	6.71^a^	6.67^a^	6.51^a^	0.06	0.771
**42th days**							
Thymus index (%)	1.32^a^	1.41^a^	1.36^a^	1.62^a^	1.73^a^	0.07	0.281
Spleen index (%)	1.00^a^	1.02^a^	1.11^a^	0.88^a^	0.98^a^	0.04	0.530
Bursa index (%)	0.80^a^	0.82^a^	1.03^a^	1.06^a^	0.82^a^	0.06	0.518
Muscular stomach *p*H	3.77^a^	3.84^a^	3.42^a^	3.78^a^	3.54^a^	0.08	0.401
Jejunum *p*H	6.28^a^	5.81^a^	5.83^a^	5.57^a^	5.78^a^	0.09	0.179
Cecal *p*H	6.74^a^	6.75^a^	7.01^a^	6.71^a^	6.89^a^	0.07	0.595

*^1^Tissues index (percent of tissues weight relative to body weight). ^2^NC, negative control, basal diet and basal drinking water with no antibiotic supplementation; PC, positive control, antibiotics supplementation; DOA, NC plus diet-administered OA supplementation; WOA, NC plus water-administered OA supplementation; MOA, NC plus diet-administered and water-administered OA supplementation. Values are expressed as means with pooled SEM values. *P*-value is expressed combined significance. In the same line, values with different letters are significantly different for all possible combinations of these different groups (*P* < 0.05 or *P* < 0.01).*

**TABLE 4:** Effects of organic acids supplementation on serum indices relative to immune response in broilers at the age of 21 and 42 days in this experiment.

**Items**	**Groups^1^**					**SEM**	***P*-value**
	**NC**	**PC**	**DOA**	**WOA**	**MOA**		
**21th days**							
IgM	4.14^a^	3.45^ab^	3.31^ab^	3.53^ab^	3.18^b^	0.16	0.308
IgA	4.68^a^	3.91^b^	2.77^c^	1.41^cd^	0.81^d^	0.24	<0.001
IgG	13.38^c^	15.33^bc^	16.52^b^	15.67^bc^	19.09^a^	0.45	0.001
Antibody titers ND	5.38^a^	4.56^a^	4.94^a^	5.00^a^	5.50^a^	0.20	0.606
**42th days**							
IgM	2.73^bc^	5.12^a^	3.19^ab^	2.01^bc^	0.88^c^	0.39	0.009
IgA	1.91^a^	1.42^b^	1.17^bc^	0.70^c^	1.03^bc^	0.09	<0.001
IgG	15.95^a^	16.72^ab^	18.39^a^	16.43^ab^	14.37^b^	0.61	0.356
Antibody titers ND	3.44^a^	3.31^a^	3.50^a^	2.81^a^	3.44^a^	0.162	0.682

*^1^NC, negative control, basal diet and basal drinking water with no antibiotic supplementation; PC, positive control, antibiotics supplementation; DOA, NC plus diet-administered OA supplementation; WOA, NC plus water-administered OA supplementation; MOA, NC plus diet-administered and water-administered OA supplementation. Values are expressed as means with pooled SEM values. *P*-value is expressed combined significance. In the same line, values with different letters are significantly different for all possible combinations of these different groups (*P* < 0.05 or *P* < 0.01).*

Compared with the NC group, AGP increased body weight (*P* = 0.002 and *P* < 0.001) at the ages of 21 and 42 days, daily weight gain (*P* = 0.002, *P* = 0.002, and *P* < 0.001), and average daily intake (*P* = 0.002, *P* < 0.001, and *P* < 0.001) during the 1–21 days, 22–42 days, and 1–42 days phases, survival rate (*P* = 0.039) during 1–42 days phase and IgM (*P* = 0.045) at the age of 42 days, and decreased feed consumption (*P* = 0.031) during the 1–21 days phase and IgA (*P* = 0.047 and *P* = 0.003) at the ages of 21 and 42 days of broilers in the PC group. Compared with the NC group, DOA increased body weight (*P* = 0.022 and *P* = 0.051) at ages of 21 and 42 days, daily weight gain (*P* = 0.021, *P* = 0.052, and *P* = 0.054) during the 1–21 days, 22–42 days, and 1–42 days phases, average daily intake (*P* = 0.040 and *P* = 0.044) during the 22–42 and 1–42 days phases, and IgG (*P* = 0.020) at the age of 21 days and decreased IgA (*P* < 0.001 and *P* = 0.003) at the ages of 21 and 42 days of broilers in the DOA group; WOA increased body weight (*P* = 0.016) at the age of 42 days, daily weight gain (*P* = 0.025 and *P* = 0.015) and average daily intake (*P* = 0.030 and *P* = 0.038) during the 22–42 days and 1–42 days phases and decreased IgA (*P* < 0.001 and *P* < 0.001) at the ages of 21 and 42 days of broilers in the WOA group; MOA increased IgG (*P* < 0.001) at the age of 21 days and decreased IgA (*P* < 0.001 and *P* = 0.001) at the ages of 21 and 42 days and IgM (*P* = 0.047) at the age of 21 days of broilers in the MOA group.

Compared with the PC group, DOA decreased body weight (*P* = 0.041) at the age of 42 days, daily weight gain (*P* = 0.040) during the 1–42 days phase, average daily intake (*P* = 0.048 and *P* = 0.052) during the 1–21 days and 1–42 days phases, and IgA (*P* = 0.001) at the age of 21 days of broilers in the DOA group; WOA increased feed consumption (*P* = 0.008) during the 1–21 days phase and decreased body weight (*P* = 0.029) at the age of 21 days, daily weight gain (*P* = 0.030), average daily intake (*P* = 0.030) during the 1–21 days phase, IgA (*P* < 0.001 and *P* = 0.005) at the ages of 21 and 42 days and IgM (*P* = 0.010) at the age of 42 days of broilers in the WOA group; MOA decreased body weight (*P* = 0.013) at the age of 42 days, daily weight gain (*P* = 0.010 and *P* = 0.014) during the 22–42 days and 1–42 days phases, average daily intake (*P* = 0.014, *P* < 0.001, and *P* < 0.001) during the 1–21 days, 22–42 days, and 1–42 days phases, feed consumption (*P* = 0.027) during the 1–42 days phase, IgA (*P* < 0.001) at the age of 21 days and IgM (*P* < 0.001) at the age of 42 days of broilers in the MOA group, and only increased IgG (*P* < 0.001) at the age of 21 days.

### Global Sequencing Data

An average of 84,404 valid sequences per sample was obtained, and an average of 79,492 high-quality sequences per sample (representing 94.23% of the valid sequences, ranging from 49,886 to 80,370) was acquired after data trimming and quality filtering. The normalized depth of 71,194 reads per sample based on downstream analyses described in the results. OTUs (97% identity) were clustered into independent species belonging to 19 phyla, 42 classes, 71 orders, 144 families, and 738 genera. All Good’s coverages were >0.99, which suggested the microbial diversity within the samples of broilers cecal chyme at the two stages in this study had been sufficiently captured.

### Changes in Cecal Microbiota Composition

#### Bacterial Composition at Phylum Level

**FIGURE 1:**
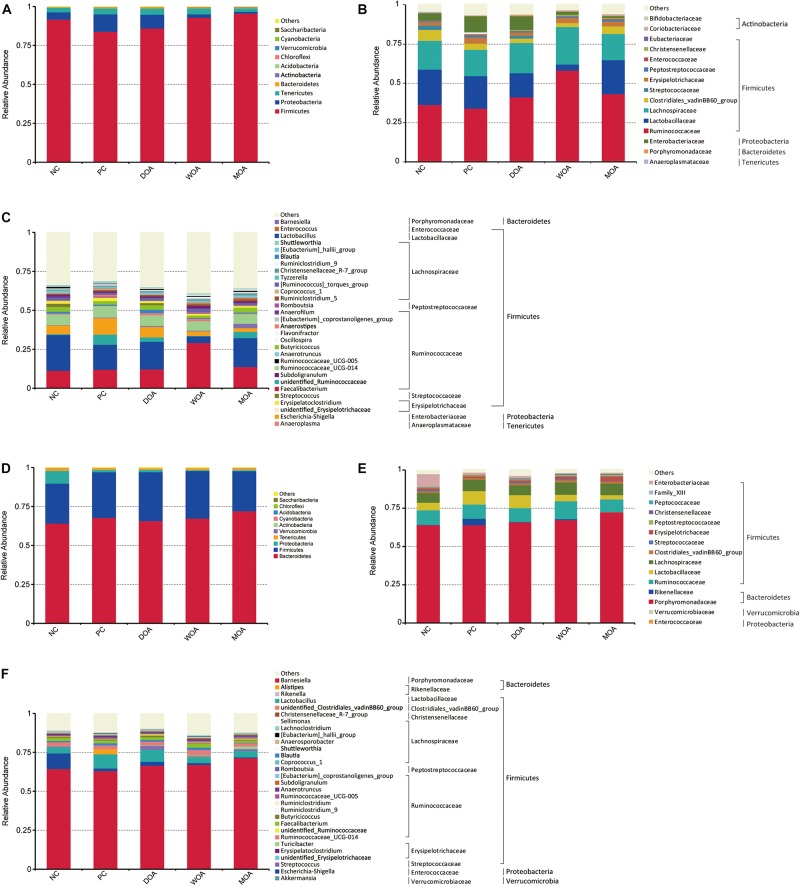
Distribution of the most abundant taxa of cecal microbiota in broilers at the ages of 21 and 42 days. **(A)** Phylum at 21 days. **(B)** Family at 21 days. **(C)** Genus at 21 days. **(D)** Phylum at 42 days. **(E)** Family at 42 days. **(F)** Genus at 42 days. Values are expressed as the means. Only taxa that occupied more than 0.5% in at least one group at the phylum level or more than 3% at the family and genus level are presented. NC, negative control, basal diet and basal drinking water with no antibiotic supplementation; PC, positive control, antibiotics supplementation; DOA, NC plus diet-administered OA supplementation; WOA, NC plus water-administered OA supplementation; MOA, NC plus diet-administered and water-administered OA supplementation.

Bacterial phyla with a relative abundance ≥0.0001% was identified in the microbiota residing in the cecal chyme of broilers at the ages of 21 and 42 days, where *Gemmatimonadetes* and *Planctomycetes* were exclusively found in the top 15. In general, microbiota in chicken cecal niches were dominated by *Firmicutes*, *Bacteroidetes*, *Proteobacteria*, *Tenericutes*, and *Actinobacteria*. From the first 1–21 days phase to the second 22–42 days phase, their proportion changed greatly. During the first 1–21 days phase, *Firmicutes* (90.50%), *Proteobacteria* (5.57%), and *Tenericutes* (2.99%) made up 99% of the cecal microbiota, and the proportion of these three phyla was reduced to 28.52, 2.51, and 1.20% (*P* < 0.001, *P* = 0.030, and *P* < 0.001), respectively, in the cecum during the 22–42 days phase, while *Bacteroidetes* was greatly increased from 0.44 to 67.35% (*P* < 0.001) (Figure 1A and Supplementary Table S1). Additionally, for the top 10 phyla, except for *Actinobacteria*, which decreased from 0.24 to 0.10% (*P* < 0.001), and *Cyanobacteria*, which increased from 0.001 to 0.026% (*P* < 0.001), there was no change found in the remaining microbial composition. From the age of 21 to 42 days, there were significant changes in the relative abundance of three or more bacterial phyla in the top 10 of each group (data not shown).

At the age of 21 days, the relative abundance at the phylum level of *Firmicutes* (top 10-1, *P* = 0.004), *Proteobacteria* (top 10-2, *P* = 0.003), *Actinobacteria* (top 10-5, *P* = 0.007), *Verrucomicrobia* (top 10-7, *P* = 0.035), and *Saccharibacteria* (top 10-10, *P* = 0.016) were significantly affected by the various treatments (Figure 1A and Supplementary Table S2). No significant effect (*P* > 0.05) in the experiment was found regarding *Bacteroidetes*, *Firmicutes*/*Bacteroidetes* (F/B), *Tenericutes*, *Cyanobacteria*, *Acidobacteria*, and *Chloroflexi*, and there was no change at the phylum level for *Gemmatimonadetes* and *Planctomycetes* (Supplementary Table S2). Compared with the NC group, *Firmicutes* was decreased at a relatively stable level close to 8% by AGP treatment (91.56:83.83%, *P* = 0.035), whereas *Proteobacteria* was increased by 2.37-fold (4.75:11.22%, *P* = 0.021), but there was no change in the level of *Actinobacteria*, *Verrucomicrobia*, and *Saccharibacteria* (*P* > 0.05). *Firmicutes* and *Proteobacteria* were not affected by the three OA treatments, whereas *Actinobacteria* was increased 3.00- and 4.07-fold by the WOA (0.103:0.329%, *P* = 0.022) and MOA treatments (0.103:0.419, *P* = 0.002), respectively, and *Verrucomicrobia* and *Saccharibacteria* were increased 3.05- and 10.80-fold, respectively, only by the DOA treatment (0.0062:0.0189%, *P* = 0.024; 0.0005:0.0054%, *P* = 0.007) (Figure 1A and Supplementary Table S2). Compared with the PC group, *Firmicutes* was increased at a relatively stable level close to 12% by the WOA (83.83:95.63%, *P* = 0.002) and MOA (83.83:95.59%, *P* = 0.002) treatments, whereas *Proteobacteria* was decreased 6.00- and 9.43-fold (11.22:1.87%, *P* = 0.002; 11.22:1.19%, *P* = 0.001), respectively, but there was not change due to DOA treatment (*P* > 0.05); *Actinobacteria*, *Verrucomicrobia*, and *Saccharibacteria* were not affected by the WOA and MOA treatments, whereas *Verrucomicrobia* and *Saccharibacteria* were increased 9.95- and 6.75-fold, respectively, only by DOA treatment (0.0019:0.0189%, *P* = 0.004; 0.0008:0.0054%, *P* = 0.010) (Figure 1A and Supplementary Table S2). The F/B of the DOA group was lower than that in the WOA group (*P* = 0.036) (Supplementary Table S2). The relative abundance of *Firmicutes* and *Actinobacteria* in the DOA group was lower than that in the WOA group (85.88:95.63%, *P* = 0.010; 0.107:0.329%, *P* = 0.025) and MOA group (85.88:95.56%, *P* = 0.010; 0.107:0.419%, *P* = 0.002), whereas the relative abundance of *Proteobacteria*, *Verrucomicrobia*, and *Saccharibacteria* was higher than that in the WOA group (8.82:1.87%, *P* = 0.014; 0.0189:0.0060%, *P* = 0.022; 0.0054:0%, *P* = 0.003) and MOA group (8.82:1.19%, *P* = 0.008; 0.0189:0.0051%, *P* = 0.015, 0.0054:0.0003%, *P* = 0.004) (Figure 1A and Supplementary Table S2). There was no difference in the relative abundance at the phylum level in the cecal chyme of broilers between the WOA and MOA groups.

At the age of 42 days, the relative abundance at the phylum level of *Proteobacteria* (top 10-3, *P* = 0.009), *Actinobacteria* (top 10-5, *P* = 0.053), *Cyanobacteria* (top 10-6, *P* = 0.033), and *Saccharibacteria* (top 10-10, *P* = 0.031) was significantly affected by the various treatments (Figure 1D and Supplementary Table S3). No significant effect (*P* > 0.05) was found regarding *Firmicutes*, *Bacteroidetes*, F/B, *Tenericutes*, *Verrucomicrobia*, *Acidobacteria*, and *Chloroflexi*, and there was no change at the phylum level of *Gemmatimonadetes* and *Planctomycetes* (Supplementary Table S3). Compared with the NC group, *Proteobacteria* and *Cyanobacteria* were decreased 5.96- and 6.00-fold, respectively, by AGP treatment (10.60:1.78%, *P* = 0.006; 0.0306:0.0051%, *P* = 0.002), whereas *Actinobacteria* and *Saccharibacteria* were increased by 1.82- and 9.59-fold, respectively (0.0592:0.1079%, *P* = 0.054; 0.00032:0.00307%, *P* = 0.017); *Proteobacteria* was affected by the three OA treatments, which were decreased 7. 79-, 16. 56-, and 17.10-fold by the DOA (10.60:1.36%, *P* = 0.005), WOA (10.60:0.64%, *P* = 0.003), and MOA (10.60:0.62%, *P* = 0.002) treatments, respectively; *Actinobacteria* and *Cyanobacteria* were affected by the WOA treatment (0.0592:0.1190%, *P* = 0.024; 0.0306:0.0153%, *P* = 0.041), whereas *Saccharibacteria* was not affected by the three OA treatments (Figure 1A and Supplementary Table S3). Compared with the PC group, only *Saccharibacteria* was affected by the three OA treatments, which were decreased 9. 59-, 307. 00-, and 307.00-fold by the DOA (0.00307:0.00032%, *P* = 0.017), WOA (0.00307:0%, *P* = 0.008), and MOA (0.00307%:0%, *P* = 0.008) treatments, respectively (Figure 1A and Supplementary Table S3). The relative abundance of *Actinobacteria* in the DOA group was lower than that in the WOA group (0.0615:0.1190%, *P* = 0.024) (Figure 1A and Supplementary Table S3). As at the age of 42 days, there was no difference in the relative abundance at the phylum level in the cecal chyme of broilers between the WOA and MOA groups.

#### Bacterial Composition at Family Level

At the family level (top 15), the relative abundance of *Enterobacteriaceae* (*P* = 0.003) within *Proteobacteria* and *Coriobacteriaceae* within *Actinobacteria* (*P* = 0.003) were significantly affected by the various treatments at the age of 21 days (Figure 1B and Supplementary Table S4), and *Enterobacteriaceae* (*P* = 0.016) within *Proteobacteria* was also affected at the age of 42 days (Figure 1E and Supplementary Table S5). Except for *Eubacteriaceae* within *Firmicutes* (*P* = 0.042) at the age of 21 days and *Streptococcaceae* (*P* = 0.033) and *Enterococcaceae* (*P* = 0.046) within *Firmicutes* at the age of 42 days, there was no difference in the relative abundance of the predominant genera at the family level in the cecal chyme of broilers between the PC group and the DOA group.

#### Bacterial Composition at Genus Level

At the genus level, the relative abundance at the predominant microbiota of *Blautia* (*P* = 0.045), *Clostridium_sensu_stricto_1*(*P* = 0.046)and *Streptococcus* (*P* < 0.001) within *Firmicutes*, *Escherichia_Shigella* (*P* = 0.001) within *Proteobacteria*, and *Adlercreutzia* (*P* = 0.011) within *Actinobacteria* was significantly affected by the compositive treatments by AGP and OAs at the age of 21 days (Figure 1C and Supplementary Table S6), and *Lactobacillus* (*P* = 0.001) and *Enterococcus* (*P* = 0.004) within Firmicutes, *Escherichia_Shigella* (*P* = 0.009) within *Proteobacteria*, and *Bifidobacterium* (*P* = 0.007) within *Actinobacteriawere* affected at the age of 42 days (Figure 1F and Supplementary Table S7). Except for *Blautia* (*P* = 0.020), *Anaerofustis* (*P* = 0.042) within *Firmicutes* at the age of 21 days and *Streptococcaceae* (*P* = 0.034) and *Enterococcus* (*P* = 0.042) within *Firmicutes*, *Bifidobacterium* within *Actinobacteria* (*P* = 0.002) at the age of 42 days, there was no difference in the relative abundance of the predominant genera at the genus level in the cecal chyme of broilers between the PC group and the DOA group. Among those symbiotic beneficial bacteria, *Bifidobacterium* and *Lactobacillus* were checked, and their levels were highest in DOA-treated or/and PC-treated broilers (Supplementary Tables S6, S7), respectively. Notably, among these potentially harmful pathogens, *Enterococcus*, *Streptococcus*, and *Escherichia_coli* (*P* = 0.007 and *P* = 0.014, Supplementary Tables S8, S9) at the species level within *Escherichia_Shigella* were found at the ages of 21 and 42 days, *Clostridium_perfringens* (*P* = 0.046, Supplementary Table S8) at the species level within *Clostridium_sensu_stricto_1* was found at the age of 21 days, and their levels were significantly affected by the compositive treatments by AGP and OAs; *Enterococcus* was highest in PC-treated and MOA-treated broilers at the age of 21 days and only in PC-treated broilers at the age of 42 days; *Streptococcus* was highest in NC-treated and DOA-treated broilers at the age of 21 days and only lowest in PC-treated broilers at the age of 42 days; *Escherichia_Shigella* was highest in NC-treated and PC-treated broilers at the age of 21 days and but only in NC-treated broilers at the age of 42 days; *Escherichia_coli* was highest in PC-treated broilers at the age of 21 days and but only in NC-treated broilers at the age of 42 days; and *Clostridium_perfringens* was highest in NC-treated broilers at the age of 21 days.

### Diversity and Richness of Microbiota in Cecal Chyme

**FIGURE 2:**
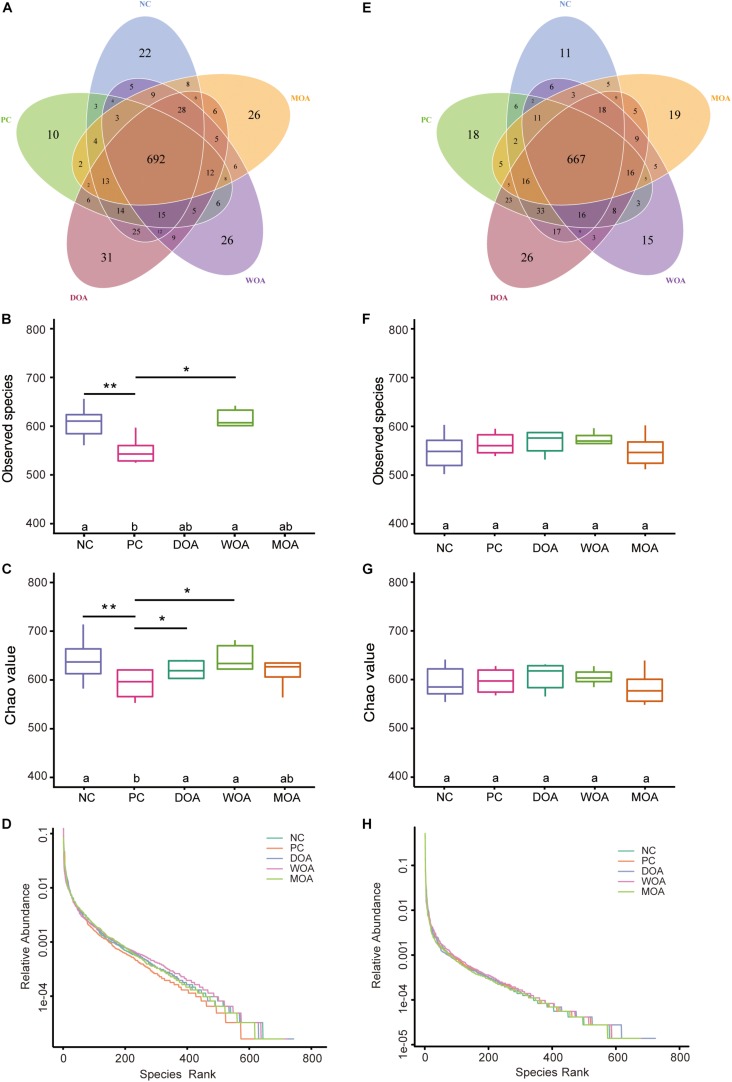
Alpha-diversity of the microbiome residing in the cecal chyme of broilers at the ages of 21 and 42 days. **(A)** Venn diagram at 21 days. **(B)** OTU values at 21 days. **(C)** Chao1 values at 21 days. **(D)** Rank abundance curves at 21 days. **(E)** Venn diagram at 42 days. **(F)** OTU values at 42 days. **(G)** Chao 1 values at 42 days. **(H)** Rank abundance curves at 42 days. In the Venn diagram, each circle represents one group, and the number of circles and circles overlapped represents the number of OTUs shared between the groups, while the number without overlap represents the number of OTUs unique to the group. The rank abundance curve is OTUs in the sample according to the relative abundance (or contain sequence number) in order of larger to smaller. To obtain the corresponding order number, take the abscissa of the OTU sort code, the relative abundance of OTUs as the ordinate, and connect these points with broken line, namely, drawing a rank abundance curve; it can intuitively reflect the richness and evenness of species in the sample. In the horizontal direction, species richness is reflected by the width of the curve; the higher the species richness is, the greater the span of the curve on the horizontal axis is. In the vertical direction, the smoothness of the curve reflects the evenness of species in the sample; the flatter the curve is, the more homogeneous the species distribution will be. NC, negative control, basal diet and basal drinking water with no antibiotic supplementation; PC, positive control, antibiotics supplementation; DOA, NC plus diet-administered OA supplementation; WOA, NC plus water-administered OA supplementation; MOA, NC plus diet-administered and water-administered OA supplementation. ^∗^ and ^∗∗^ indicates a significant difference at the 0.05 or 0.01 level between the two groups that were compared.

Numbers of OTUs, Shannon index, Simpson index, ACE, chao1, Good’s coverage, PD-whole-tree index, and rank abundance curve indicating alpha diversity of microbiota were substantially increased and showed higher diversity and richness in the cecal chyme of broilers at the age of 21 days than at 42 days in this experiment. The number of OTUs shared between the five groups at the age of 21 and 42 days was 692 and 667, respectively (Figures 2A,E). The numbers of OTUs, ACE, chao1, PD-whole-tree index, and rank abundance curve showed lower microbiota homogenization and richness in the cecal chyme of PC-treated broilers than in those of NC-, DOA-, or WOA-treated broilers at the age of 21 days, especially DOA-treated broilers (Figures 2B–D and Supplementary Table S10). However, at the age of 42 days, alpha diversity parameters were not affected by the various treatments (Figures 2F–H and Supplementary Table S11), and the rank abundance curve (Figure 2H) showed mildly higher microbiota homogenization and richness in the cecal chyme of DOA-treated broilers than in those of the other groups.

**FIGURE 3:**
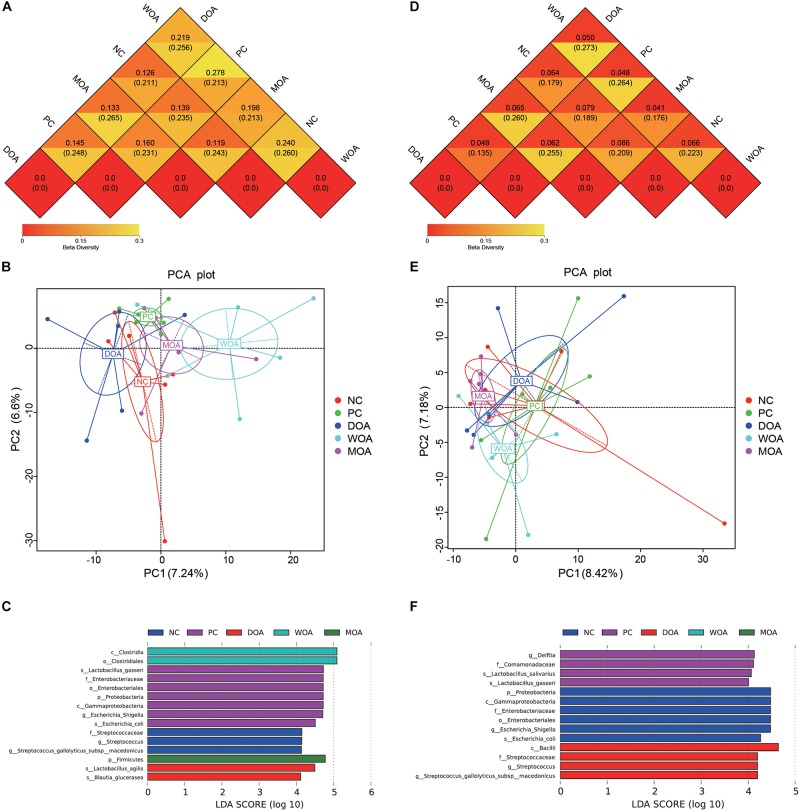
Beta-diversity of microbiome residing in the cecal chyme of broilers at the ages of 21 and 42 days. **(A)** Beta-diversity heatmap at 21 days. **(B)** PCA plot at 21 days. **(C)** LDA distribution histogram at 21 days. **(D)** Beta-diversity heatmap at 42 days. **(E)** PCA plot at 42 days. **(F)** LDA distribution histogram at 42 days. Values are expressed as the means. NC, negative control, basal diet and basal drinking water with no antibiotic supplementation; PC, positive control, antibiotics supplementation; DOA, NC plus diet-administered OA supplementation; WOA, NC plus water-administered OA supplementation; MOA, NC plus diet-administered and water-administered OA supplementation. p, phylum; c, class; o, order; f, family; g, genus; s, species. In the beta-diversity heatmap, the two values in the same grid represent the distance of the weighted UniFrac distance and the unweighted UniFrac distance, and the number in the grid is the difference coefficient between two groups; the smaller the difference coefficient is, the smaller the difference in species diversity is. In PCA plots, axes represent the two dimensions explaining the greatest proportion of variances in the communities for each analysis; if the community composition of groups is more similar, the distance between them in PCA diagram is closer. The LDA value distribution histogram shows the species whose LDA score is greater than the set value (the default setting is 4), that is, the biomarker with a significant difference between the groups. The length of the histogram represents the influence of different species (LDA score).

Beta diversity was assessed by (un)weighted UniFrac distance analysis and principal component analysis (PCA). Unweighted and weighted UniFrac distance analysis showed that microbiota diversity parameters were affected by the various treatments at the age of 21 days (unweighted *P* = 0.048; weighted *P* = 0.009) and were not affected by the various treatments at the age of 42 days (unweighted *P* = 0.429; weighted *P* = 0.225). It was clear that the cecal species diversity of DOA-treated broilers was closer to that of either NC-treated or PC-treated broilers at the age of 21 days than that of MOA-treated broilers; that of WOA broilers separated from that of the treated broilers in other groups (Figures 3A,B). Interestingly, weighted UniFrac distance analysis showed that the cecal microbiota compositions of broilers in these five groups were not separated (Figure 3D), but the weighted UniFrac distances showed that the cecal microbiota of DOA-treated broilers was still closer to that of either NC-treated or PC-treated broilers at the age of 42 days (Figures 3D,E).

Respective permutational multivariate ANOVA (Adonis), analysis of similarities (ANOSIM), and multiple response permutation procedure (MRPP) *P*-values showing the statistical significance of community variation among these different treatments at the ages of 21 and 42 days are indicated (Table 5). The results confirmed the structural dissimilarity in the cecal microbiota community between NC-treated and PC-treated broilers or between PC-treated and OA-treated broilers at the age of 21 days. However, there was no structural dissimilarity in the cecal microbiota community of broilers in these five groups at the age of 42 days. More specifically, the kinds of the species with significant differences in abundance (LDA score ≥ 4) in the NC-, PC-, DOA-, WOA-, and MOA-treated groups at the age of 21 days were 3, 7, 2, 2, and 1 (Figure 3C), and at the age of 42 days were 6, 4, 4, 0, and 0 (Figure 3F), respectively.

**TABLE 5:** Adonis, MRPP, and ANOSIM *P*-values based on microbial community between groups.

**vs. groups^1^**	**21 days**			**42 days**		
	**Adonis *P*-values**	**MRPP *P*-values**	**ANOSIM *P*-values**	**Adonis *P*-values**	**MRPP *P*-values**	**ANOSIM *P*-values**
NC vs. PC	0.003	0.010	0.008	0.412	0.420	0.300
NC vs. DOA	0.281	0.267	0.308	0.684	0.775	0.718
NC vs. WOA	0.014	0.007	0.030	0.315	0.273	0.247
NC vs. MOA	0.215	0.253	0.147	0.147	0.177	0.204
PC vs. DOA	0.031	0.048	0.016	0.462	0.577	0.500
PC vs. WOA	0.002	0.003	0.005	0.367	0.428	0.447
PC vs. MOA	0.001	0.004	0.003	0.111	0.247	0.312
DOA vs. WOA	0.010	0.009	0.143	0.924	0.903	0.819
DOA vs. MOA	0.137	0.181	0.146	0.560	0.558	0.404
WOA vs. MOA	0.031	0.040	0.098	0.504	0.512	0.715

*^1^NC, negative control, basal diet and basal drinking water with no antibiotic supplementation; PC, positive control, antibiotics supplementation; DOA, NC plus diet-administered OA supplementation; WOA, NC plus water-administered OA supplementation; MOA, NC plus diet-administered and water-administered OA supplementation. Adonis, permutation multivariate analysis of variance; MRPP, multiple response permutation procedure; ANOSIM, analysis of similarities.*

### Microbiome Responding to Growth Performance, Tissue Index, Chyme pH, and Serum Indices

**FIGURE 4:**
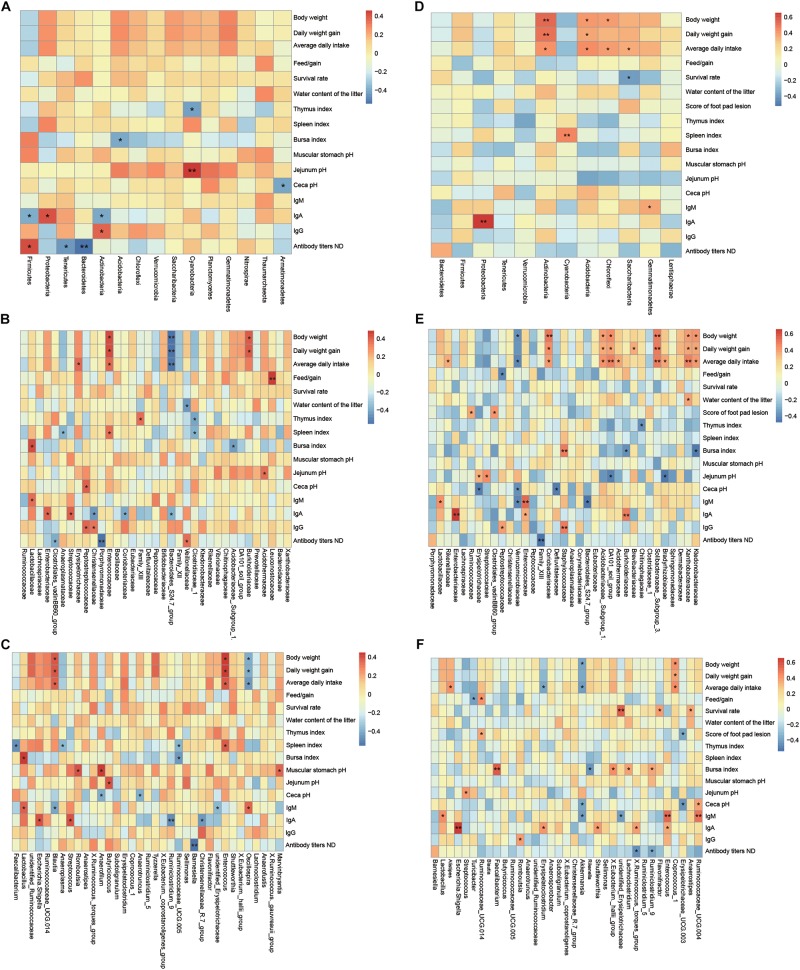
Correlation between the most abundant taxa of the cecal microbiota at the level of phylum, family, and genus and growth performance, gut chyme pH, tissue index, and serum immune response indices of broilers at the ages of 21 and 42 days. **(A)** Phylum at 21 days. **(B)** Family at 21 days. **(C)** Genus at 21 days. **(D)** Phylum at 42 days. **(E)** Family at 42 days. **(F)** Genus at 42 days. The values corresponding to the intermediate heat map are Spearman correlation coefficients *r*, which is between −1 and +1, negative correlation when *r* < 0, and positive correlation when *r* > 0; results of significance tests were *P* < 0.05 or *P* < 0.01 when marked ^∗^ or ^∗∗^, respectively. Only taxa that occupied more than 0.5% in at least one region at the phylum level or more than 3% at the family and genus level are presented.

The correlation between the dominant taxon of cecal microbiota at the level of phylum, family, and genus levels relative to growth performance, gut chyme pH, tissue index, serum indices, and immune response of broilers at the ages of 21 and 42 days was observed and assessed by Spearman rank correlation analysis (Figure 4). At the age of 21 days, body weight (*P* = 0.046), daily weight gain (*P* = 0.050), average daily intake (*P* = 0.017), spleen index (*P* < 0.001), bursa index (*P* = 0.005), and antibody titers NDA (*P* = 0.056) screened out by CCA-envfit function analysis (Supplementary Table S12) were environmental factors that have a more significant impact on the bacterial community; *Lachnospiraceae*, *Enterococcaceae*, *Erysipelotrichaceae*, and *Lactobacillaceae* within *Firmicutes*, *Enterobacteriaceae* and *Burkholderiaceae* within *Proteobacteria* were positively correlated with these indices; *Ruminococcaceae* within *Firmicutes*, *Porphyromonadaceae Bacteroidales_S24.7_group* within *Bacteroidetes*, and *Anaeroplasmataceae* within *Tenericutes* were negatively correlated with these indices (Figures 4A–C). At the age of 42 days, body weight (*P* = 0.038), daily weight gain (*P* = 0.007), feed/gain ratio (*P* = 0.050), and jejunum pH (*P* = 0.025) screened out by CCA-envfit function analysis (Supplementary Table S13) were environmental factors that had a more significant impact on the bacterial community; *Lachnospiraceae*, *Erysipelotrichaceae*, *Streptococcaceae*, and *Ruminococcaceae* within *Firmicutes*, *Rikenellaceae* within *Bacteroidetes*, *Bradyrhizobiaceae* and *Xanthobacteraceae* within *Proteobacteria*, *Ktedonobacteraceae* within *Chloroflexi*, *DA101_soil_group* within *Verrucomicrobia*, *Acidothermaceae*, *Brevibacteriaceae*, *Solibacteraceae_Subgroup_3*, and *Coriobacteriaceae* within *Actinobacteria* were positively correlated with these indices; and *Erysipelotrichaceae* within *Firmicutes*, *Verrucomicrobiaceae* within *Verrucomicrobia*, and *Bradyrhizobiaceae* within *Proteobacteria* were negatively correlated with these indices (Figures 4D–F).

### Predicted Gene Functions of the Microbiota

**FIGURE 5:**
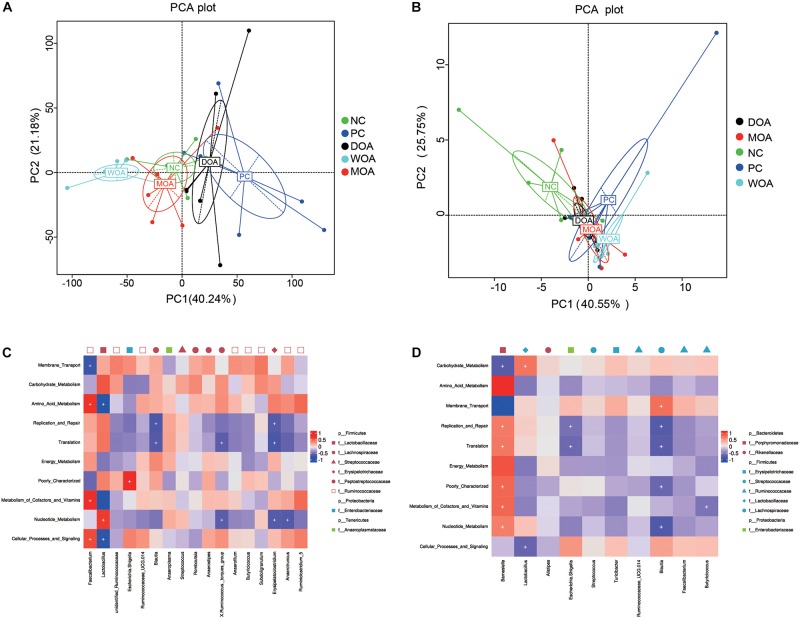
Principal component analyses (PCAs) of gene functions of the microbiome residing in ceca at the age of 21 days **(A)** and at the age of 42 days **(B)**. Heatmap illustrating correlations (red: positive; blue: negative) between phylogenetic groups at the level of genus (the core OTU in the top 10) and predicted gene functions (the level 2 categories in the top 10) for the cecal microbiome at the age of 21 days **(C)** and at the age of 42 days **(D)**. NC, negative control, basal diet and basal drinking water with no antibiotic supplementation; PC, positive control, antibiotics supplementation; DOA, NC plus diet-administered OA supplementation; WOA, NC plus water-administered OA supplementation; MOA, NC plus diet-administered and water-administered OA supplementation. As long as there is a group with an average abundance greater than 1%, the OTU is selected as the core OTU. Only the categories of predicted gene functions that occupied more than 1.0% of metagenomes in at least one group annotated with KEGG pathway analysis at the KO hierarchy level 2 are presented. Significant correlations are indicated by “+” (*P* < 0.05).

To understand the functional differences among cecal microbiome, PCAs revealed a cluster of NC and MOA and PC and DOA, which was clearly separated from WOA at the age of 21 days (Figure 5A); other clusters of four groups together at the age of 42 days are shown in Figure 5B, and WOA only crossed with MOA. The predominant gene functions, including membrane transport (*P* < 0.001, *P* = 0.033), amino acid metabolism (*P* = 0.023, *P* = 0.002), energy metabolism (*P* = 0.136, *P* = 0.013), cellular processes and signaling (*P* = 0.054, *P* = 0.544), and poorly characterized function (*P* = 0.005, *P* = 0.010), were significantly affected by the various treatments at the ages of 21 days (Supplementary Table S14) and 42 days (Supplementary Table S15). At the age of 21 days, comparing with the NC-treated group, the number of different predominant gene functions of the PC-, DOA-, WOA-, or MOA-treated groups was 3, 1, 1, or 0, respectively; comparing with the PC-treated group, the number of different predominant gene functions of the DOA-, WOA-, or MOA-treated groups was 0, 6, or 2, respectively; five of the top 10 gene functions differed between the DOA-treated and WOA-treated groups; zero of the 10 gene functions differed between the DOA-treated and MOA-treated groups or between the WOA-treated and MOA-treated groups. At the age of 42 days, comparing with the NC-treated group, the number of different predominant gene functions of the PC-, DOA-, WOA-, or MOA-treated groups was 5, 4, 7, or 4, respectively; there was no difference in these predominant gene functions between the PC-, DOA-, WOA-, or MOA-treated groups; one of the 10 gene functions differed between the DOA-treated and WOA-treated groups; and two of the 10 gene functions differed between the DOA-treated and MOA-treated groups; there was no difference in these predominant gene functions between the WOA-treated and MOA-treated groups.

The correlation between microbial gene functions and intestinal OTUs presented similar patterns in at the ages of 21 days (Figure 5C) and 42 days (Figure 5D). At the age of 21 days, *Faecalibacterium* within *Firmicutes* was positively correlated with gene functions related to the metabolism of energy, amino acids, vitamins and cofactors, and cellular processes and signaling and negatively correlated with membrane transport, but *Lactobacillus* within *Firmicutes* presented an adverse correlation; and *Blautia*, *X_Ruminococcus._torques_group*, *Erysipelatoclostridium*, and *Anaerotruncus* within *Firmicutes* were negatively correlated with gene functions related to replication and repair, translation, and nucleotide metabolism; *Escherichia_Shigella* within *Proteobacteria* was positively correlated with poorly characterized gene functions.

At the age of 42 days, *Barnesiella* within *Bacteroidetes* was positively correlated with gene functions related to translation, amino acid metabolism, metabolism of cofactors and vitamins, replication and repair, energy metabolism, poorly characterized functions, and nucleotide metabolism and negatively correlated with carbohydrate metabolism and membrane transport; *Lactobacillus* within *Firmicutes* was positively correlated with gene functions related to carbohydrate metabolism and negatively correlated with cellular processes and signaling; *Escherichia_Shigella* within *Proteobacteria* was negatively correlated with replication and repair and translation; *Blautia* within *Firmicutes* was positively correlated with gene functions related to membrane transport and negatively correlated with translation, replication and repair, nucleotide metabolism, and poorly characterized functions; and *Butyricicoccus* within *Firmicutes* was negatively correlated with metabolism of cofactors and vitamins.

## Discussion

### A Better Alternative Starts Its Growth-Promoting Function Early and Is More Welfare to the Animal Than AGPs in Terms of Final Weight

As in some previous studies, the two kinds of commercially available OA supplementation of diet or water in this present study are promising non-antibiotic alternatives to Virginiamycin in broiler feed, which have shown positive effects on improving final weight, average daily gain, and average daily intake (Ruhnke et al., 2015; Polycarpo et al., 2017; Rodjan et al., 2018), without harmfully affecting growth performance, such as feed utilization, survival rate, welfare index (foot pad lesion and litter moisture), tissue (including thymus, spleen, and bursa) index, and gut (including muscular stomach, jejunum, and ceca) chyme pH. DOA can be utilized as a better performance enhancement than WOA during the starter stage; the final results were slightly lower than those found with antibiotics and were not different from WOA. However, it was noteworthy that feed supplemented with MOA (mixed DOA and WOA) did not affect growth performance, tissue index, or gut chyme pH in broiler chickens. DOA as a better alternative, its growth-promoting functions start earlier than WOA, and the presented final weigh slightly less than Virginiamycin may be more friendly on animal welfare.

The composition of intestinal microbiota is important for maintaining homeostasis of the gastrointestinal tract and the health of the host (Zhang et al., 2018). The intestinal microbiota is a complex ecosystem with dynamic diversity that shifts with diet and time (Isaacson and Kim, 2012). It was confirmed that the gastrointestinal tract in vertebrate was rapidly colonized by a complex microbial community during the neonatal period. In mammals, there is accumulating evidence that interference with early intestinal microbial colonization can have long-lasting beneficial or harmful health effects on individuals (Benis et al., 2015; Korpela and de Vos, 2018; Valles and Francino, 2018). In order to have an insight into how microbial consortium are assembled, it is important to understand the forces that shape the early community, especially during the nursing stage (Frese et al., 2015; Gresse et al., 2017). However, data on intestinal microbiota from birth in broilers using modern molecular methods are scarce, and fewer experimental studies have examined the differential effects of AGPs and its alternatives on microbiota consortium in chicken cecal niches (Costa et al., 2017). In this study, sub-therapeutic antibiotics and OAs as AGP alternatives were used to supplement broilers from birth and to create a food animal model. Three kinds of growth phenotype had presented after DOA, WOA, and MOA (mixed DOA and WOA) supplementation. Then, 16S rDNA sequencing from the cecal chyme was used for community analysis of gut microbiota at two different timepoints to detect the reaction to these forces.

### A Better Alternative Provides a Similar and Better Selective Force Than AGPs on Microbiome Composition in the Cecum of Broilers in the Early Growth Stage

*Firmicutes* + *Proteobacteria* + *Tenericutes* and *Bacteroidia* + *Firmicutes* + *Proteobacteria* dominated at the phylum level in the cecal chyme of broilers at the age of 21 and 42 days, respectively, constituting greater than 98% of the microbiome. Indeed, previous studies on the effects of Virginiamycin on intestinal microbiome of broilers were lacking during different growth stages (Pourabedin et al., 2015; Costa et al., 2017). At the ages of 21 and 42 days, among these three OA groups, the composition and abundance of cecal microbial dominant species in broilers in the DOA-treated group were the closest to those of the AGP group at the levels of phylum, family, and genus, Especially in the relative abundance of *Proteobacteria* and *Firmicutes* of broilers at the age of 21 days.

### Selection on *Proteobacteria*

It was known that *Proteobacteria* include some zoonotic pathogens and many other notable pathogenic microbia (Mancabelli et al., 2016; Gresse et al., 2017; Clavijo and Florez, 2018). Compared with the NC group, as shown in this study, Virginiamycin and three OAs significantly decreased the relative abundance of *Proteobacteria* by 5.96–17.10 times in the cecum of broilers aged 42 days, and this was accompanied by a decrease in the genus *Escherichia_Shigella* within *Enterobacteriaceae*. However, AGP did not consistently exert a positive inhibiting effect on *Proteobacteria*; at 21 days of age, Virginiamycin increased the relative abundance of *Proteobacteria* by 2.37 times and was accompanied by an increase in the genus *Escherichia_Shigella*, suggesting that AGP had a time-dependent effect on the abundance of *Proteobacteria*. This finding is supported by Looft et al. (2012) and Salaheen et al. (2017) who reported that sub-therapeutic concentrations of penicillin, chlortetracycline and sulfamethazine administered to piglets and of tylosin, neomycin sulfate, bacitracin, erythromycin, and oxytetracycline administered to broilers increased the prevalence of *Proteobacteria*. The three OAs present a positive effect on *Proteobacteria* at two different timepoints, and this shift was driven by unaltered or decreased relative abundance of *Escherichia_shigella* at the genus level populations and *Escherichia_coli* at the species level populations. With the increase of acidic material feeding, the reduced extent of Escherichia was increased. Among them, DOA has no effect on the relative abundance of *Proteobacteria* at age of 21 days, showing no difference from the AGP group. Unfortunately, MOA treatment with higher OA concentrations had no role in the growth of broiler chickens. This finding seems to contradict previous works suggesting that the blend of medium-chain OAs (10% malic acid, 13% citric acid, and 17% fumaric acid) (Nguyen et al., 2018), the blend of short-chain OAs (15% propionic acid, 24% formic acid) (Owens et al., 2008), and 4% gluconic acid (Biggs and Parsons, 2008) could decrease *Escherichia_coli* abundance and simultaneously increase body weight gain. Multiple factors, including differences in species, kinds of OAs, the experimental designs, or blends may contribute to the divergence of the results. The results suggested that Virginiamycin had the opposite effect on the relative abundance of *Proteobacteria* during the early and later stages of broiler development. Short-chain OAs could play the substitute role of AGP in promoting growth through effective interference with the relative abundance of *Proteobacteria*; moreover, the addition of DOA provided a similar and better selective force than AGPs for the colonization of *Proteobacteria* in the cecum of broiler chickens in the early growth stage.

### Selection on Firmicutes

Compared with the NC group, as shown in this study, Virginiamycin extremely significantly decreased the relative abundance of *Firmicutes* in the cecum of broilers aged 21 days, and this shift was accompanied by an extremely significant decrease in *Clostridium_perfringens* and increases in *Anaerofustis* by several times. *Clostridium_perfringens* could cause enteritic gas gangrene in animals and food poisoning in humans (Uzal et al., 2015; Navarro et al., 2018) and *Anaerofustis* has been proved to be a kind of good bacteria that can resist inflammation (Arrazuria et al., 2016; Wu et al., 2018). Meanwhile, unlike previous reports (Singh et al., 2013; Salaheen et al., 2017), the relative abundance of *Lactobacillus* was stable, which is beneficial for animal growth performance (Fajardo et al., 2012; Rodriguez-Lecompte et al., 2012); but the relative abundance of *Lactobacillus_gasseri* (shown in Supplementary Table S8) increased by 9.26 times and 9.90 times in Virginiamycin treated cocks at the age of 21 and 42 days, which could involve in some pathogenic strain specific anti-proliferative activity (Kobayashi et al., 2017; Sungur et al., 2017) and enhance immunoregulation followed by periodontitis prevention in mucosa via the gut immune system (Kobayashi et al., 2017). The persistent increased *Lactobacillus_gasseri* may be why Virginiamycin have consistently positive effect on weight gain. It was reported (Singh et al., 2013; Salaheen et al., 2017) that an increased F/B ratio accompanying increased *Lactobacillus* was associated with penicillin and a blend of Oxytetracycline, Erythromycin, Tylosin, Bacitracin, and Neomycin as AGP supplementation in broilers, no change in the mean F/B ratio between the NC and PC groups was observed in this study. In contrast with the NC treatment, treatment with in this study presented no effect on the relative abundance of *Firmicutes*, but the relative abundance of *Firmicutes* in the WOA and MOA groups was higher than in the PC group. Profitably, the three OAs significantly decreased the relative abundance of *Clostridium_perfringens*, but WOA decreased the relative abundance of *Lactobacillus* and increased the relative abundance of *Faecalibacterium* to maintain intestinal health throughout life (Lopez-Siles et al., 2017; Maier et al., 2017). The previous work reported that 4% gluconic acid reduced the cecal populations of *Lactobacillus* and *Clostridium_perfringens* and had no effects on growth performance in New Hampshire × Columbian cocks at the age of 21 days (Biggs and Parsons, 2008). The reduced Lactobacillus may be why WOA and MOA have no effect on weight gain at the age of 21 days. Until the age of 42 days, AGPs and OAs had no effect on the relative abundance of *Firmicutes* and the F/B ratio. The addition of Virginiamycin kept the relative abundance of *Anaerofustis* relatively high, which may be why the effects of Virginiamycin on growth performance were slightly higher than those found with OAs. In contrast to Virginiamycin and DOA treatment enriching *Lactobacillus* and *Anaerofustis*, the WOA and MOA treatments decreased or did not alter the relative abundance of them, which may be why the MOA treatment had no effect on final weight gain. It was suggested that different kinds of non-therapeutic antibiotics and potential alternatives did not have any consistent effects on the intestinal microbial composition of broilers in different stages and/or varieties. The results suggested that the addition of DOA provided a similar selective force as AGPs for the colonization of *Firmicutes* in the cecum of broiler chickens in the early and later growth stage.

### Selection on Some Beneficial and Pathogenic Bacteria

*Streptococcus* was identified as representative of usage of Virginiamycin (Costa et al., 2017). It was generally believed that Streptogramin could fight and Inhibit Multidrug-resistant *staphylococcus*, *Streptococcus*, and *Enterococcus* that were resistant to methicillin or vancomycin (Allington and Rivey, 2001). Noticeably, Virginiamycin could decrease *Clostridium_sensu_stricto_1* and *Streptococcus* (Asam et al., 2015; Dumke et al., 2015), and but increased *Enterococcus* (Wang et al., 2015; Beukers et al., 2017) at the age of 21 days, which were as a commensal of both food animal and human gastrointestinal tracts and associated with malignant clinical infection of gastrointestinal tract in animals. In contrast, the three OAs also could eliminate the colonization of *Clostridium_sensu_stricto_1*, DOA and WOA treatments had no effect on the final relative abundance of *Enterococcus* and *Streptococcus*, and but MOA treatment as same as Virginiamycin treatment increased the relative abundance of *Enterococcus* at the age of 21 days. It was suggested that there was too high of a concentration of OAs during the early growth stage and Virginiamycin could not restrain the early colonization of *Enterococcus* but could restrain the colonization of *Streptococcus*. It is surprising that *Barnesiella* was increased by DOA treatment at the age of 21 days, which enabled clearance of intestinal antibiotic-resistant *Enterococcus* colonization (Ubeda et al., 2013). As a typical probiotic, a transient *Bifidobacterium* (Bottacini et al., 2017; Million et al., 2017; Yang et al., 2017) bloom was influenced by DOA supplementation at the age of 42 days, which can effectively inhibit pathogens associated with severe acute malnutrition and improve the metabolic capacity of the host digestive tract. The high abundance colonization of these two types of bacteria may be one of the reasons why DOA promotes the growth of broiler chickens. Furthermore, it cannot be ignored that at the age of 21 days, Virginiamycin and DOA treatment could decrease the relative abundance of *Oscillospira*, which is positively associated with leanness (Konikoff and Gophna, 2016; Gophna et al., 2017), and *Anaeroplasma*, which is an intracellular bacteria of cells of hematopoietic origin and an etiological agent of tick-borne diseases (Battilani et al., 2017). In short, the interference of DOA treatment with the early colonization of probiotics and pathogens in the broiler cecum is the closest to AGP treatment, and OAs did not cause the occurrence of Virginiamycin-resistant strains of *Enterococcus*.

*Enterococcus*, *Streptococcus*, and *Lactobacillus* belong to *Lactobacillales*. The commensal presence of *Lactobacillus* and these pathogens potentially stimulates the immunoprotection of the intestinal mucosal barrier (Li et al., 2017; Korpela and de Vos, 2018; Zhang et al., 2018). However, the overgrowth of pathogens microbes could disturb barrier function and consequently result in enteric diseases. In humans, it was demonstrated that antibiotic utilization could shift some kinds of microbiota away from the normal developmental pattern (Li et al., 2017; Korpela and de Vos, 2018), and disruptions alter the microbial signals and then potentially affect host development. In previous *in vitro* coculture studies, a selective bias toward probiotic populations (e.g., *Firmicutes*, specifically *Lactobacillus*), was confirmed in the presence of antibiotics in broth, milk, and/or chicken fecal medium, and/or the supplementation of AGP alternatives when cocultured with pathogens (Deng et al., 2015; Peng et al., 2015; Salaheen et al., 2016; Sharma et al., 2017). However, *in vivo* microbial composition is complex, and due to competitive inhibition and/or symbiotic promotion, there may be large differences in the final relative abundances of these intestinal microbial species under different selective pressures, such as different kinds of OAs (Czerwinski et al., 2010; Goodarzi et al., 2014). Although AGP supplementation increased the relative abundance of *Escherichia* at the age of 21 days, with particular lack of effect on *Enterococcus*, this phenomenon did not affect the persistent growth-promoting function of AGPs and even exceeded the ability of some OAs. In the process of evaluating the impact of antibiotics and their alternatives on the intestinal microbiota of food animals, their influences on one or some bacteria cannot be simply characterized, but the adjustment of the entire bacterial ecology needs to be comprehensively considered from all levels (Benis et al., 2015; Li et al., 2017; Schokker et al., 2017).

### A Better Alternative Provides Better Selective Force Than AGPs on Microbiome Richness, Homogenization, and Diversity in the Cecum of Broilers in the Early Growth Stage

The abundance of OTUs, colonization area, and presence or absence of OTUs contributed to the diversity of the microbiota in the cecum (Zhang et al., 2018), which is positively correlated with intestinal health. Inducing homogenization of the intestinal microbiota has been proposed one mechanism of actions of AGP for promoting both growth and growth uniformity in farm animals (Collier et al., 2003; Nava et al., 2009). There was no difference in the alpha-diversity and beta-diversity of microbial species in the cecal chyme of broilers in each treatment group at the age of 42 days, but the microbial ecology formed under these early-life perturbations during the initiation stage presented significant differences. It was same as that the overall gut microbial diversity of Ross at the age of 35 days (Pourabedin et al., 2015) or Cobb 500 at the age of 43 days (Costa et al., 2017) was not affected by treatment with Virginiamycin (16.5 mg/kg). As the previous study (Heinsen et al., 2015), the molecular ecological data of alpha-diversity at the age of 21 days indicate that Virginiamycin decreased OTU numbers and homogenization of species distribution. The OAs might have a mechanism of action that is distinct with respect to antibiotics. Bacterial populations of DOA and WOA-treated birds were more homogeneous and richer than those of birds treated with AGPs, and the alpha-diversity of microbial community in DOA group was closer to that in NC group. In this study, the results showed that Virginiamycin significantly decreased the observed microbial species in the broiler cecum. Significant differences in cecal microbial beta-diversity were found between the NC group and PC group as well as between the PC group and any group of the three OA treatments, although these differences did not exist between the NC and DOA groups.

Interestingly, WOA supplementation presented an uncanny resemblance to promoting effects on final weight gain but separated species diversity and predicted gene functions of cecal microbiota in treated broilers distinctly from those of treated broilers in the NC, PC, and DOA groups, especially during the early growth stage. Among the three OA groups, the diversity of microbial community in DOA group is more similar to that in NC group and PC group, and there were dominant bacteria species, respectively. Specifically, the increased *Barnesiella* (Ubeda et al., 2013) and *Blautia* (Jenq et al., 2015) by DOA supplementation equipped the cecum with indispensable metabolic capabilities for host survival. The increased *Verrucomicrobia* (Derrien et al., 2017; Fujio-Vejar et al., 2017), *Saccharibacteria* (Opdahl et al., 2018; Starr et al., 2018), and *Cyanobacteria* (Raja et al., 2016; Wang et al., 2016) due to DOA supplementation at the age of 21 days could be more involved in the metabolism, digestion of nutrients, and immune function. The increased and a persistent increase in *Coriobacteriaceae* (Ogawara, 2015; Landwehr et al., 2016) was caused by WOA treatment, which may be one of the reasons for the growth of broilers. These results suggest that different OAs play a different role in promoting growth by interfering with the colonization of intestinal microorganisms, as well as Virginiamycin.

### A Better Alternative Provides a Similar Selective Force With AGPs on Microbial Metabolism Functions in the Cecum of Broilers Especially in the Early Growth Stage

Metabolic diversions were indicated by predicted gene functions of the microbiome residing in broilers ceca that were provided AGP or DOA supplementation, although both of them resulted in higher body weight in chickens. These differences can be explained by the variability in microbiota. In particular, *Barnesiella* certified the presence of numerous carbohydrate-active enzymes to hydrolyze the cytoderm components from plant-based diets (Thomas et al., 2011), which were increased by DOA supplementation at the age of 21 days and significantly associated with serum immune indices (e.g., ND antibody titers). Four aspects were analyzed comprehensively, including bacteria with a significantly different proportion between groups, environmental factors that have a significant influence on the bacterial community, bacteria with a significant correlation with the environmental factors, and predicted metabolic functions that are enriched as determined by KEGG pathway analysis. Notably, in this study, some bacteria were significantly associated with body growth and immune indices (e.g., IgM), including *Oscillospira* and *Blautia* that were modified by DOA supplementation and were negatively correlated with metabolism of replication and repair and translation; *Lactobacillus* associated with serum immune indices and immune tissue growth, was modified by Virginiamycin and DOA supplementation, and was correlated with gene functions related to replication and repair, translation, and nucleotide metabolism; and *Enterococcaceae* associated with serum immune indices (e.g., IgM and IgA), was modified by Virginiamycin and OA supplementation, and was positively correlated with poorly characterized gene functions.

Corresponding to the selected and separated microbial diversity, the predicted gene functions of the microbiota in the WOA group broiler chickens differed significantly from those of the PC or DOA groups at the age of 21 days and those of the NC group at the age of 42 days. However, no explanation is currently available for increased body weight due to WOA supplementation that was observed in the present study. It was presumed that an increase of *Christensenellaceae* as an obese-associated microbiome constituent (Biagi et al., 2016; Bottacini et al., 2017) and *Faecalibacterium* as an activator of Toll-like receptor 2 (Maier et al., 2017) and the persistent increase of *Actinobacteria* that is used for screening novel anti-infectives (Ogawara, 2015; Landwehr et al., 2016) and significantly associated with body growth or spleen growth in this study may involve an increasing abundance of dominant species in their cooccurrence network to affect the later development of broiler chickens. MOA treatment with higher OA concentrations had no promoting effect on growth performance or the composition, diversity, and predicted gene functions of cecal microbial communities in broilers and, as well as Virginiamycin, could not restrain the early colonization of *Enterococcus* but could restrain the colonization of *Streptococcus* during the whole experiment. The observed results are consistent with the proposed hypothesis that temporary early-life changes in the composition and/or diversity of intestinal microbiota modified by the differential selection of antibiotics and their alternatives may programmatically induce long-lasting effects on intestinal digestive and immune systems, which may lead to different growth phenotype in later stage (Benis et al., 2015; Li et al., 2017; Schokker et al., 2017). It is suggested that adding AGPs and its alternatives in early life development or special window period may achieve the effect of adding AGPs and its alternatives in the whole growth process.

In practice, it has been confirmed that DOAs markedly modify bacterial composition in crops, and feed expansion increased *Lactobacillus* and lactate in crops (Heres et al., 2004; Goodarzi et al., 2014). The periodical and pH-quantitative preparation of WOAs before drinking may increase the labor costs and the difficulty in cleanliness of the potable water devices, and feed expansion and pellet-borne delivery may result in loss of OAs. Considering that the effects of DOA supplementation on growth performance and immune relative indices were the closest to that of AGP supplementation, the effects of DOA treatment on the homogenization and species diversity of cecal dominant microbial community were better than that of AGP supplementation, and OAs with higher concentration could restrain the colonization of *Streptococcus*, therefore after appropriately increasing the amount of supplementation, DOA supplementation would be a more suitable alternative to sub-therapeutic Virginiamycin. However, the effective addition period of DOA remains to be studied.

## Conclusion

In summary, by taking advantage of 16S rDNA sequencing, this study obtains a topographical map of the broiler microbiome in cecal chyme. Heterogeneities and cooccurrence in microbial assembly, structure, and function are noted in broiler ceca, responding to supplemented antibiotics or OAs as AGP alternatives. Establishment of the consortium of microbes with better diversity and assembly of the early dominant microbial community with similar populations were involved in the growth performance effects of supplemented OAs as AGPs alternatives in broilers. The presented results underscored the importance of intestine early-life microbial colonization in relation to broiler growth development, and suggested the importance of the research on the effective addition time of new AGPs or AGPs alternatives. Simultaneously, such a comprehensive perspective may provide a theoretical basis for how to select, evaluate, and improve AGPs alternatives in food animals.

## Data Availability Statement

All datasets generated for this study are included in the article/Supplementary Material.

## Ethics Statement

This study was carried out in accordance with the guidelines of the Animal Ethics Committee of the Chinese Academy of Agricultural Sciences. The protocol was approved by the Administration of Affairs Concerning Experimental Animals (The State Science and Technology Commission of China, 1988).

## Author Contributions

SS, LW, YHu, YW, and YHa conceived and designed the experiments. LW, QW, and DS performed the experiments. YHu and LW analyzed the data. YHu prepared the manuscript. YHu and SS revised the manuscript.

## Conflict of Interest

YW and YHa were employed by Trouw Nutrition R&D.

The remaining authors declare that the research was conducted in the absence of any commercial or financial relationships that could be construed as a potential conflict of interest.
