# Supplementary material for: Selectived and Reshaped Early Dominant Microbial Community in the Cecum With Similar Proportions and Better Homogenization and Species Diversity Due to Organic Acids as AGP Alternatives Mediate Their Effects on Broilers Growth
Source: Front Microbiol. 2020 Jan 14;10:2948. doi: 10.3389/fmicb.2019.02948 (PMC6971172; doi:10.3389/fmicb.2019.02948)
Supplement: Supplementary file 1 [file Table_1.docx]

Table S1. Comparison of the relative abundance (%) of the predominant microbiota at the phyla level in the cecal digesta of broilers at the age of 21 days and 42 days in this experiment.

| Taxonomy | 21days | 42 days | SEM | *P* value |
| --- | --- | --- | --- | --- |
| Firmicutes | 90.50 | 28.52 | 4.17 | < 0.001 |
| Proteobacteria | 5.57 | 2.51 | 0.71 | 0.030 |
| Bacteroidetes | 0.44 | 67.35 | 4.44 | < 0.001 |
| Tenericutes | 2.99 | 1.20 | 0.27 | < 0.001 |
| Actinobacteria | 0.243 | 0.102 | 0.021 | < 0.001 |
| Acidobacteria | 0.0150 | 0.0202 | 0.0030 | 0.393 |
| Chloroflexi | 0.0052 | 0.0071 | 0.0011 | 0.389 |
| Verrucomicrobia | 0.008 | 0.076 | 0.022 | 0.112 |
| Saccharibacteria | 0.00141 | 0.00171 | 0.00042 | 0.721 |
| Cyanobacteria | 0.0006 | 0.0263 | 0.0035 | < 0.001 |
| Gemmatimonadetes | 0.00076 | 0.00119 | 0.00023 | 0.357 |
| Planctomycetes | 0.00060 | 0.00011 | 0.00018 | 0.172 |
| Others | 0.237 | 0.184 | 0.033 | 0.427 |

Notes: Superscript 1: NC = negative control, no antibiotics; PC = positive control, antibiotics included; DOA = NC plus diet-tranmission OA; WOA = NC plus drinking-water type OA; MOA = NC plus diet-tranmission OA and drinking-water type OA. Values are expressed as means with pooled SEM values. In the same line, values with different letters are significantly different for all possible combinations of these different groups (*P* < 0.05 or *P* < 0.01).
