# Supplementary material for: Selectived and Reshaped Early Dominant Microbial Community in the Cecum With Similar Proportions and Better Homogenization and Species Diversity Due to Organic Acids as AGP Alternatives Mediate Their Effects on Broilers Growth
Source: Front Microbiol. 2020 Jan 14;10:2948. doi: 10.3389/fmicb.2019.02948 (PMC6971172; doi:10.3389/fmicb.2019.02948)
Supplement: Supplementary file 9 [file Table_9.docx]

Table S9. Effects of Virginiamycin and organic acids supplementation on the relative abundance (%) of the predominant microbiota at the species classification in the cecal digesta of broilers at the age of 42 days.

| Taxonomy | | | | Groups ^1^ | | | | | SEM | *P* value |
| --- | --- | --- | --- | --- | --- | --- | --- | --- | --- | --- |
| Phyla classification | Family classification | Genus classification | Species classification | NC | PC | DOA | WOA | MOA |  |  |
| Bacteroidetes | Bacteroidaceae | Bacteroides | Bacteroides_fragilis | 0.00037 ^a^ | 0.00037 ^a^ | 0.00112 ^a^ | 0 ^a^ | 0.00112 ^a^ | 0.00024 | 0.498 |
|  | Porphyromonadaceae | Barnesiella | Barnesiella_viscericola | 5.01 ^a^ | 4.80 ^a^ | 5.08 ^a^ | 4.55 ^a^ | 5.26 ^a^ | 0.19 | 0.828 |
| Firmicutes | Bacillaceae | Bacillus | Bacillus_alcalophilus | 0 ^a^ | 0 ^a^ | 0 ^a^ | 0 ^a^ | 0.00074 ^a^ | 0.00015 | 0.426 |
|  | Aerococcaceae | Aerococcus | Aerococcus_viridans | 0 ^a^ | 0.00149 ^b *^ | 0.00037 ^ab^ | 0.00074 ^ab^ | 0 ^a #^ | 0.00023 | 0.054 |
|  | Enterococcaceae | Enterococcus | Enterococcus_durans | 0.077 ^ab^ | 0.121 ^b^ | 0.041 ^a #^ | 0.023 ^a #^ | 0.013 ^a ##^ | 0.013 | 0.605 |
|  | Lactobacillaceae | Lactobacillus | Lactobacillus_agilis | 1.08 ^a^ | 0.24 ^a^ | 0.80 ^a^ | 0.86 ^a^ | 0.44 ^a^ | 0.18 | 0.605 |
|  |  |  | Lactobacillus_crustorum | 0.005 ^a^ | 0.007 ^a^ | 0.006 ^a^ | 0.001 ^a^ | 0 ^a^ | 0.002 | 0.553 |
|  |  |  | Lactobacillus_gasseri | 0.20 ^a^ | 1.98 ^b **^ | 0.07 ^a ##^ | 0.07 ^a ##^ | 0.06 ^a ##^ | 0.20 | 0.002 |
|  |  |  | Lactobacillus_mucosae | 0.0011 ^a^ | 0 ^a^ | 0.0015 ^a^ | 0 ^a^ | 0 ^a^ | 0.0003 | 0.244 |
|  |  |  | Lactobacillus_pontis | 0.1 ^a^ | 0 ^a^ | 3.9 ^a^ | 0 ^a^ | 0.1 ^a^ | 0.8 | 0.438 |
|  |  |  | Lactobacillus_salivarius | 1.19 ^ab^ | 2.74 ^b^ | 0.60 ^a ##^ | 1.13 ^a #^ | 0.80 ^a #^ | 0.27 | 0.090 |
|  |  |  | Lactobacillus_vaginalis | 0.009 ^a^ | 0.020 ^a^ | 0.064 ^a^ | 0.013 ^a^ | 0.014 ^a^ | 0.011 | 0.547 |
|  |  |  | Lactococcus_garvieae | 0.0037 ^a^ | 0.0022 ^a^ | 0.0037 ^a^ | 0 ^a^ | 0 ^a^ | 0.0010 | 0.630 |
|  | Streptococcaceae | Streptococcus | Streptococcus_gallolyticus_subsp._macedonicus | 0.6 ^b^ | 0 ^a *^ | 2.6 ^b #^ | 0.7 ^b #^ | 0.1 ^b ##^ | 0.4 | 0.197 |
|  | Christensenellaceae | Christensenellaceae_R-7_group | bacterium_YE57 | 0.0007 ^a^ | 0.0030 ^a^ | 0.0007 ^a^ | 0.0022 ^a^ | 0.0026 ^a^ | 0.0004 | 0.205 |
|  | Eubacteriaceae | Eubacterium | Eubacterium_limosum | 0 ^a^ | 0 ^a^ | 0 ^a^ | 0 ^a^ | 0.0078 ^a^ | 0.0016 | 0.426 |
|  | Lachnospiraceae | [Ruminococcus]_gauvreauii_group | bacterium_ic1296 | 0.046 ^b^ | 0.039 ^b^ | 0.039 ^ab^ | 0.061 ^b^ | 0.017 ^a * ##^ | 0.005 | 0.042 |
|  |  | [Ruminococcus]_torques_group | bacterium_ic1294 | 0.00074 ^a^ | 0.00074 ^a^ | 0.00037 ^a^ | 0 ^a^ | 0 ^a^ | 0.00019 | 0.563 |
|  |  | Anaerosporobacter | Clostridiales_bacterium_77-5d | 0 ^a^ | 0 ^a^ | 0.25 ^a^ | 0 ^a^ | 0 ^a^ | 0.05 | 0.423 |
|  |  | Anaerostipes | Anaerostipes_butyraticus | 0.014 ^a^ | 0.034 ^a^ | 0.026 ^a^ | 0.063 ^a^ | 0.040 ^a^ | 0.009 | 0.579 |
|  |  | Blautia | Blautia_glucerasea | 0.93 ^ab^ | 1.34 ^ab^ | 1.26 ^ab^ | 1.17 ^b^ | 0.24 ^a^ | 0.24 | 0.609 |
|  |  | Lachnoclostridium | Clostridium_sp._BR31 | 0.078 ^a^ | 0.083 ^a^ | 0.088 ^a^ | 0.091 ^a^ | 0.073 ^a^ | 0.009 | 0.976 |
|  |  | Roseburia | Clostridia_bacterium_UC5.1-2E3 | 0.098 ^a^ | 0.028 ^a^ | 0.027 ^a^ | 0.029 ^a^ | 0.139 ^a^ | 0.021 | 0.322 |
|  |  | Sellimonas | bacterium_P1C11 | 0.122 ^a^ | 0.105 ^a^ | 0.117 ^a^ | 0.135 ^a^ | 0.078 ^a^ | 0.016 | 0.869 |
|  | Peptococcaceae | Desulfotomaculum | Desulfotomaculum_ruminis | 0.00074 ^ab^ | 0 ^a^ | 0.00149 ^b #^ | 0 ^a^ | 0 ^a^ | 0.00022 | 0.125 |
|  | Ruminococcaceae | Anaerotruncus | Anaerotruncus_colihominis | 0.0026 ^a^ | 0.0041 ^a^ | 0.0037 ^a^ | 0.0063 ^ab^ | 0.0108 ^b *#^ | 0.0011 | 0.141 |
|  |  | Butyricicoccus | Butyricicoccus_pullicaecorum | 0.094 ^b^ | 0.035 ^a *^ | 0.039 ^a *^ | 0.049 ^ab^ | 0.038 ^a *^ | 0.009 | 0.224 |
|  |  | Candidatus_Soleaferrea | Ruminococcaceae_bacterium_GD6 | 0 ^a^ | 0 ^a^ | 0.00037 ^a^ | 0.00037 ^a^ | 0.00223 ^b ** ##^ | 0.00024 | 0.006 |
|  |  | Faecalibacterium | bacterium_ic1379 | 0.49 ^b^ | 0.13 ^ab^ | 0.05 ^a *^ | 0.09 ^ab^ | 0.32 ^ab^ | 0.07 | 0.208 |
|  |  | Ruminiclostridium_9 | Clostridiales_bacterium_NK3B98 | 0.0030 ^a^ | 0.0037 ^a^ | 0.0071 ^a^ | 0.0033 ^a^ | 0.0045 ^a^ | 0.0013 | 0.887 |
|  |  | Subdoligranulum | Subdoligranulum_variabile | 0.00037 ^a^ | 0 ^a^ | 0 ^a^ | 0.00149 ^b * ##^ | 0 ^a^ | 0.00019 | 0.038 |
|  |  | unidentified_Ruminococcaceae | Ruminococcaceae_bacterium_AM2 | 0.030 ^a^ | 0.016 ^a^ | 0.066 ^a^ | 0.019 ^a^ | 0.023 ^a^ | 0.009 | 0.413 |
|  |  |  | Ruminococcus_sp._16442 | 0.0052 ^a^ | 0.0097 ^a^ | 0.0082 ^a^ | 0.0104 ^a^ | 0.0059 ^a^ | 0.0013 | 0.676 |
|  | Erysipelotrichaceae | Erysipelatoclostridium | [Clostridium]_spiroforme | 0.101 ^a^ | 0.133 ^a^ | 0.108 ^a^ | 0.071 ^a^ | 0.063 ^a^ | 0.022 | 0.870 |
|  |  |  | bacterium_ic1391 | 0.25 ^a^ | 0.23 ^a^ | 0.10 ^a^ | 0.11 ^a^ | 0.05 ^a^ | 0.05 | 0.647 |
|  |  | Faecalitalea | [Eubacterium]_tortuosum | 0 ^a^ | 0.00037 ^a^ | 0 ^a^ | 0 ^a^ | 0 ^a^ | 0.00007 | 0.426 |
|  |  | unidentified_Erysipelotrichaceae | Clostridiales_bacterium_10-3b | 0.072 ^a^ | 0.040 ^a^ | 0.026 ^a^ | 0.044 ^a^ | 0.021 ^a^ | 0.010 | 0.513 |
|  | Veillonellaceae | Megasphaera | Clostridiales_bacterium_60-7e | 0.036 ^ab^ | 0.028 ^a^ | 0.092 ^ab^ | 0.196 ^b #^ | 0.156 ^ab^ | 0.027 | 0.210 |
|  |  |  | Megasphaera_elsdenii | 0 ^a^ | 0 ^a^ | 0.00074 ^a^ | 0 ^a^ | 0 ^a^ | 0.00015 | 0.426 |
| Proteobacteria | Bradyrhizobiaceae | Bradyrhizobium | Bradyrhizobium_elkanii | 0.0019 ^ab^ | 0.0048 ^ab^ | 0.0063 ^b^ | 0 ^a^ | 0 ^a^ | 0.0009 | 0.055 |
|  | Burkholderiaceae | Burkholderia-Paraburkholderia | Burkholderia_symbiont_of_Cavelerius_saccharivorus | 0.00037 ^a^ | 0.00074 ^a^ | 0.00186 ^a^ | 0 ^a^ | 0 ^a^ | 0.00032 | 0.338 |
|  | Enterobacteriaceae | Escherichia-Shigella | Escherichia_coli | 4.7 ^b^ | 0.9 ^a **^ | 1.0 ^a **^ | 0.3 ^a **^ | 0.4 ^a **^ | 0.5 | 0.014 |
|  |  | Proteus | Proteus_mirabilis | 0.0015 ^a^ | 0.0004 ^a^ | 0.0033 ^a^ | 0.0011 ^a^ | 0.0004 ^a^ | 0.0006 | 0.583 |
| Acidobacteria | unidentified_Subgroup_2 | unidentified_Subgroup_2 | bacterium_Ellin7505 | 0.00037 ^a^ | 0.00037 ^a^ | 0 ^a^ | 0.00037 ^a^ | 0 ^a^ | 0.00012 | 0.736 |
|  | Acidobacteriaceae_(Subgroup_1) | unidentified_Acidobacteriaceae_(Subgroup_1) | bacterium_Ellin326 | 0 ^a^ | 0 ^a^ | 0.00037 ^a^ | 0 ^a^ | 0.00112 ^a^ | 0.00023 | 0.507 |
|  | unidentified_Acidimicrobiales | unidentified_Acidimicrobiales | bacterium_enrichment_culture_clone_auto73_4W | 0 ^a^ | 0.00037 ^a^ | 0 ^a^ | 0 ^a^ | 0 ^a^ | 0.00007 | 0.426 |
|  | Coriobacteriaceae | Gordonibacter | Gordonibacter_pamelaeae | 0.004 ^a^ | 0.008 ^ab^ | 0.004 ^ab^ | 0.024 ^b *^ | 0.005 ^ab^ | 0.003 | 0.231 |
|  |  |  | Gordonibacter_urolithinfaciens | 0.0030 ^ab^ | 0.0082 ^ab^ | 0.0033 ^ab^ | 0.0167 ^b^ | 0.0019 ^a^ | 0.0024 | 0.246 |
|  | Brevibacteriaceae | Brevibacterium | Brevibacterium_epidermidis | 0.0048 ^a^ | 0.0041 ^a^ | 0.0019 ^a^ | 0.0019 ^a^ | 0.0030 ^a^ | 0.0008 | 0.691 |
|  | Dermabacteraceae | Brachybacterium | Brachybacterium_paraconglomeratum | 0.0019 ^a^ | 0.0030 ^a^ | 0.0037 ^a^ | 0.0045 ^a^ | 0.0011 ^a^ | 0.0007 | 0.574 |
|  | Micrococcaceae | Glutamicibacter | Glutamicibacter_arilaitensis | 0.0022 ^a^ | 0.0015 ^a^ | 0.0011 ^a^ | 0.0004 ^a^ | 0.0004 ^a^ | 0.0004 | 0.594 |
| Verrucomicrobia | unidentified_OPB35_soil_group | unidentified_OPB35_soil_group | bacterium_Ellin5102 | 0.0004 ^a^ | 0.0007 ^a^ | 0.0022 ^a^ | 0.0011 ^a^ | 0 ^a^ | 0.0004 | 0.417 |
|  | Verrucomicrobiaceae | Akkermansia | Akkermansia_muciniphila | 0.14 ^a^ | 0 ^a^ | 0.01 ^a^ | 0 ^a^ | 0.20 ^a^ | 0.04 | 0.464 |
| Gemmatimonadetes | Gemmatimonadaceae | Gemmatimonas | bacterium_Ellin5290 | 0 ^a^ | 0.00037 ^a^ | 0.00037 ^a^ | 0 ^a^ | 0 ^a^ | 0.00010 | 0.567 |
| Others | | | | 84.4 ^a^ | 86.9 ^a^ | 83.4 ^a^ | 90.1 ^b *^ | 91.3 ^b *^ | 1.0 | 0.025 |

Notes: Superscript 1: NC = negative control, basal diet and basal drinking water with no antibiotic supplementation; PC = positive control, antibiotics supplementation; DOA = NC plus diet-administered OA supplementation; WOA = NC plus water-administered OA supplementation; MOA = NC plus diet-administered and water-administered OA supplementation. Values are expressed as means with pooled SEM values. P value is expressed combined significance. In the same line, values with different letters are significantly different for all possible combinations of these different groups (*P* < 0.05 or *P* < 0.01), n = 8. In the same line, superscript * indicates significant difference between PC, DOA, WOA or MOA group and NC group at the level of 0.05, and superscript ** indicates significantly different between PC, DOA, WOA or MOA group and NC group at the level of 0.01; superscript # indicates significant difference between DOA, WOA or MOA group and NC group at the level of 0.05, and superscript ## indicates significantly different between DOA, WOA or MOA group and NC group at the level of 0.01.
