# Supplementary material for: Selectived and Reshaped Early Dominant Microbial Community in the Cecum With Similar Proportions and Better Homogenization and Species Diversity Due to Organic Acids as AGP Alternatives Mediate Their Effects on Broilers Growth
Source: Front Microbiol. 2020 Jan 14;10:2948. doi: 10.3389/fmicb.2019.02948 (PMC6971172; doi:10.3389/fmicb.2019.02948)
Supplement: Supplementary file 13 [file Table_13.docx]

Table S13. Effects of environmental factors on microbiome distribution by CCAenvfit analysis in the cecal digesta of broilers at the age of 42 days in this experiment.

| Environmental factors | CCA1 | CCA2 | r2 | Pr ( > r) |
| --- | --- | --- | --- | --- |
| Body weight | -0.865 | -0.502 | 0.220 | 0.038 |
| Daily weight gain | -0.842 | -0.539 | 0.282 | 0.007 |
| Average daily intake | -0.942 | -0.336 | 0.092 | 0.276 |
| Feed/gain | 0.550 | 0.835 | 0.177 | 0.050 |
| Survival rate | 0.687 | 0.727 | 0.044 | 0.527 |
| Water content of the litter | -0.924 | 0.381 | 0.128 | 0.166 |
| Score of foot pad lesion | 0.813 | -0.582 | 0.088 | 0.224 |
| Thymus index | -0.994 | -0.110 | 0.090 | 0.304 |
| Spleen index | -0.666 | -0.746 | 0.006 | 0.888 |
| Bursa index | -0.215 | -0.977 | 0.033 | 0.570 |
| Muscular stomach pH | 0.334 | 0.942 | 0.114 | 0.169 |
| Jejunum pH | 0.402 | -0.916 | 0.212 | 0.025 |
| Ceca pH | -0.670 | -0.742 | 0.014 | 0.822 |
| IgM | -0.989 | 0.148 | 0.033 | 0.731 |
| IgA | -0.043 | 0.999 | 0.037 | 0.469 |
| IgG | -0.009 | -1.000 | 0.162 | 0.106 |
| Antibody titers ND | 0.394 | -0.919 | 0.067 | 0.370 |

Note: the values corresponding to CCA1 and CCA2 are the cosine values of the Angle between the arrow of environmental factor and the sorting axis, indicating the correlation between environmental factor and the sorting axis. R2 denotes the determination coefficient of environmental factors on species distribution, and the smaller r2 means the smaller impact of environmental factors on species distribution. Pr indicates the significance test of correlation.
