# Supplementary material for: Selectived and Reshaped Early Dominant Microbial Community in the Cecum With Similar Proportions and Better Homogenization and Species Diversity Due to Organic Acids as AGP Alternatives Mediate Their Effects on Broilers Growth
Source: Front Microbiol. 2020 Jan 14;10:2948. doi: 10.3389/fmicb.2019.02948 (PMC6971172; doi:10.3389/fmicb.2019.02948)
Supplement: Supplementary file 15 [file Table_15.docx]

Table S15. Comparison of the ratio of predicted predominant gene functions (the top 10 ratio) among microbiome residing in the cecal digesta of broilers at the age of 42 days in this experiment

| Gene Function | Groups ^1^ | | | | | SEM | *P* value |  |
| --- | --- | --- | --- | --- | --- | --- | --- | --- |
|  | NC | PC | DOA | WOA | MOA |  |  |  |
| Carbohydrate metabolism (%) | 10.69 ^b^ | 10.96 ^a^ | 10.83 ^ab^ | 10.92 ^ab^ | 10.82 ^ab^ | 0.04 | 0.307 | |
| Membrane transport (%) | 11.58 ^a^ | 10.26 ^b^ | 10.79 ^ab^ | 10.26 ^b^ | 10.47 ^b^ | 0.16 | 0.033 | |
| Replication and repair (%) | 9.44 ^b^ | 9.88 ^ab^ | 9.96 ^a^ | 9.96 ^a^ | 9.87 ^ab^ | 0.07 | 0.129 | |
| Translation (%) | 8.91 ^b^ | 9.36 ^a^ | 9.34 ^a^ | 9.40 ^a^ | 9.27 ^ab^ | 0.10 | 0.115 | |
| Amino acid metabolism (%) | 8.47 ^c^ | 8.63 ^b^ | 8.58 ^bc^ | 8.71 ^ab^ | 8.79 ^a^ | 0.03 | 0.002 | |
| Nucleotide metabolism (%) | 4.09 ^b^ | 4.24 ^ab^ | 4.27 ^a^ | 4.26 ^a^ | 4.22 ^ab^ | 0.03 | 0.213 | |
| Energy metabolism (%) | 4.10 ^b^ | 4.17 ^ab^ | 4.10 ^b^ | 4.20 ^a^ | 4.19 ^a^ | 0.01 | 0.013 | |
| Metabolism of cofactors and vitamins (%) | 3.07 ^a^ | 3.08 ^a^ | 3.04 ^a^ | 3.10 ^a^ | 3.09 ^a^ | 0.01 | 0.352 | |
| Cellular processes and signaling (%) | 1.54 ^a^ | 1.51 ^a^ | 1.48 ^a^ | 1.49 ^a^ | 1.56 ^a^ | 0.02 | 0.544 | |
| Poorly characterized (%) | 1.11 ^a^ | 1.02 ^b^ | 1.04 ^b^ | 0.99 ^b^ | 1.01 ^b^ | 0.01 | 0.010 | |

Notes: Superscript 1: NC = negative control, basal diet and basal drinking water with no antibiotic supplementation; PC = positive control, antibiotics supplementation; DOA = NC plus diet-borne OA supplementation; WOA = NC plus water-borne OA supplementation; MOA = NC plus diet-borne and water-borne OA supplementation. Values are expressed as means with pooled SEM values. In the same line, values with different letters are significantly different for all possible combinations of these different groups (*P* < 0.05 or *P* < 0.01).
